# Repurposing dronedarone induces ferroptosis through GPX4 inactivation and degradation in pancreatic cancer

**DOI:** 10.1186/s13046-026-03687-6

**Published:** 2026-03-14

**Authors:** Yanmei Gu, Yingying Wang, Gangli Liu, Rong Cui, Chenzhe Ma, Jianrong Wang, Xing Gao, Yang Zhao, Yumin Li

**Affiliations:** 1 The Second Clinical Medical College, Lanzhou University Second Hospital, Lanzhou University, Lanzhou, Gansu 730000 China; 2 School of Life Sciences, Lanzhou University, Lanzhou, Gansu 730000 China; 3 Gansu Provincial Key laboratory of Environmental Oncology, Lanzhou University, Lanzhou, Gansu 730000 China

**Keywords:** Dronedarone, High-throughput screening, Ferroptosis, Autophagy, Pancreatic cancer

## Abstract

**Background:**

Pancreatic cancer is an aggressive malignancy with poor prognosis and frequent resistance to standard anticancer therapies, highlighting the need for treatments that more effectively induce tumor cell death. Here, we aimed to identify a drug candidate that triggers cell death and to uncover its molecular mechanism in pancreatic cancer.

**Methods:**

We performed high-throughput screening using a library of 2,051 FDA-approved compounds in pancreatic cancer cells and used cell viability assays to identify agents that induce cell death. The antitumor effects of the lead compound were characterized by transmission electron microscopy, mCherry-EGFP-LC3B, lipid peroxidation and mitochondrial function assays, and an orthotopic LSL-Kras^G12D/+^, LSL-Trp53^R172H/+^, Pdx1-Cre (KPC) mouse model. Single-cell RNA sequencing, surface plasmon resonance (SPR), co-immunoprecipitation, and western blotting were used to elucidate the underlying mechanism.

**Results:**

High-throughput screening identified the antiarrhythmic agent dronedarone as a compound that significantly inhibited pancreatic cancer cell growth and can exert effective antitumor effects in vivo. Single-cell RNA sequencing and pharmacologic rescue experiments indicated that ferroptosis is a major form of dronedarone-induced regulated cell death in pancreatic cancer cells. Mechanistically, dronedarone directly bound to glutathione peroxidase 4 (GPX4), inhibited its enzymatic activity, and promoted p62-mediated autophagic degradation of GPX4, leading to mitochondrial lipid peroxidation and GPX4-dependent ferroptosis. Moreover, the ferroptosis inhibitor ferrostatin-1 attenuated dronedarone-induced lipid peroxidation, preserved GPX4 expression, and partially reversed its antitumor effects in the orthotopic KPC mouse model.

**Conclusions:**

Dronedarone serves as an unrecognized promoter of ferroptosis in pancreatic cancer by targeting p62-mediated autophagic degradation and functional inactivation of GPX4, providing a mechanistic and translational rationale for exploiting ferroptosis in pancreatic cancer.

**Supplementary Information:**

The online version contains supplementary material available at 10.1186/s13046-026-03687-6.

## Introduction

Pancreatic cancer is one of the most aggressive gastrointestinal malignancies and is associated with a dismal prognosis, which is largely due to its non-specific symptoms and insidious onset. Despite the introduction of novel chemotherapeutic agents, targeted therapy, and immunotherapies into clinical practice, drug resistance and metastasis still severely limit the effectiveness of these treatment regimens [1]. Moreover, resistance to apoptosis is a hallmark of cancer [2]. Accordingly, non-apoptotic cell death mechanisms, such as ferroptosis, may provide alternative strategies to eliminate pancreatic cancer cells. Of note, several Food and Drug Administration (FDA)-approved therapeutic agents have been characterized as ferroptosis inducers, including auranofin [3], warfarin [4] and sulfasalazine [5]. These clinically available drugs support the feasibility of translating ferroptosis-related research into clinical applications.

In recent years, ferroptosis has attracted increasing attention as an antitumor strategy driven by uncontrolled lipid peroxidation. It is characterized by accumulation of intracellular ferrous ions, elevated reactive oxygen species (ROS), and shrunken mitochondria, distinguishing it from other forms of cell death [6, 7]. Glutathione peroxidase 4 (GPX4) is a key antioxidant enzyme that, in a glutathione (GSH)-dependent manner, reduces lipid peroxides to non-toxic lipid alcohols and thereby prevents ferroptosis [8]. Consequently, targeting GPX4 to promote lipid peroxide accumulation represents a promising anticancer approach. Both enzymatic inactivation and protein degradation of GPX4 are hallmarks of ferroptosis, and the functional role of GPX4 has been implicated in tumor development and resistance to therapy [9, 10]. In this context, published evidence has shown that ferroptosis could promote therapeutic sensitivity in pancreatic cancer cells, thereby augmenting tumor-suppressive effects and making ferroptosis an attractive research topic in the field.

Dronedarone was identified from a library of FDA-approved compounds as a candidate with potential anticancer activity. Previous studies have reported that dronedarone suppresses tumor growth in several cancer types [11–13]. However, its potential antitumor mechanisms remain poorly defined. Here, we found that dronedarone induced ferroptosis and reduced cell viability in pancreatic cancer cells. Mechanistically, dronedarone increased intracellular ROS levels and directly bound to GPX4, resulting in inhibition of its enzymatic activity and subsequent p62-mediated autophagic degradation, thereby inducing ferroptosis. Furthermore, we evaluated the safety and efficacy of dronedarone in preclinical pancreatic cancer models. Our findings highlight ferroptosis as a promising therapeutic target and support the potential repurposing of dronedarone for pancreatic cancer.

## Materials and methods

### Cell culture

Pancreatic cancer cell lines (AsPC-1 and PANC-1; FUHENG Biology, China) were maintained in RPMI-1640 medium supplemented with 10% fetal bovine serum (OriCell, China) at 37 °C in a humidified incubator with 5% CO_2_. Human cardiomyocyte AC16 cells were cultured in DMEM medium (Gibco, USA) under the same incubation conditions.

### Drug screening and compounds

A library containing 2051 FDA-approved compounds was procured from MedChemExpress (USA). Compounds were first diluted to 10 µM in medium and added to 96-well plates seeded with 1,000 cells per well. After incubation for 24 h, cell viability was measured using Cell Counting Kit-8 (CCK-8) assays, and OD 450 was read using a FlexStation 3 microplate reader (Molecular Devices, USA). Primary hits were re-screened using the same procedure to confirm reproducibility. The LIVE/DEAD Viability / Cytotoxicity Kit (R37601, Thermo Fisher Scientific, USA) was applied to quantify cell viability and death in pancreatic cancer cells treated with compounds that passed rescreening, and fluorescence images were captured with a fluorescence microscope (IX73 + DP74, Olympus, Japan).

### Antibodies and reagents

Primary antibodies against GPX4 (ab125066), SQSTM1/p62 (ab207305), LC3B (ab192890) and Ki67 (ab92742) were from Abcam (USA). Antibodies against F4/80 (#28463) and Arginase-1 (#66129) were purchased from Proteintech (China). Dronedarone (HYA0016), necrostatin-1 (Nec-1, HY15760), chloroquine (CQ, HY17589A), rapamycin (Rapa, HY10219), and ferrostatin-1 (Fer-1, HY100579) were obtained from MedChemExpress (USA). Necrosulfonamide (NSA, S8251), Z-VAD-FMK (S7023) and disulfiram (DSF, S1680) were purchased from Selleck Chemicals (USA).

### Flow cytometry

AsPC-1 and PANC-1 cells were treated with dronedarone for 24 h, harvested and prepared for flow cytometric analysis. For cell-cycle analysis, cells were stained using a cell cycle staining kit (70-CCS012, MULTI SCIENCES, China) for 15 min in the dark. Apoptosis was detected with APC Annexin V Apoptosis Detection Kit (AP105, MULTI SCIENCES, China) according to the manufacturer’s instructions. Samples were acquired on a BD FACSLyric™ flow cytometer (BD Biosciences, USA).

### Animal experimentation and magnetic resonance imaging (MRI)

To generate an orthotopic xenograft model, wild-type PANC-1 cells (1 × 10^6^) were implanted into the pancreas of 8-weeks-old female NOD-SCID mice. On day 7, mice received dronedarone by intraperitoneal (50 mg/kg) every 4 days. To investigate the effect of dronedarone on ferroptosis in vivo and prepared for single-cell RNA sequencing (scRNA-seq), we established an orthotopic pancreatic cancer model based on KPC mice [LSL-Kras^G12D/+^, LSL-Trp53^R172H/+^, and Pdx1-Cre (KPC)]. KPC mice (Cat. No. NM-KI-210096; Shanghai Model Organisms Center, China) served as the tumor source. Genotypes were confirmed as described previously [14]. Briefly, tumors from KPC mice were harvested and surgically implanted into the pancreas of C57BL/6 recipient mice. One week after modeling, tumor formation was confirmed by MRI. Mice were then randomized to receive intraperitoneal injection of dronedarone, either alone or in combination with Fer-1 (5 mg/kg, twice a week). Tumor burden was monitored by MRI. Tumor length and width were measured from MRI images to calculate tumor volume as (length × width^2^)/ 2. Mice were euthanized when tumor size reaching 1500 mm^3^. All animal procedures were approved by the institutional animal care and use committee and were conducted in accordance with institutional guidelines. Mice were maintained under specific pathogen-free conditions on a 12-h light/dark cycle.

In vivo imaging was used to track tumor development. T2-weighted images were acquired on a 9.4 T MRI scanner (uMR 9.4 T, United Imaging Life Science Instrument, China) using a rapid spin-echo sequence with a repetition time = 3,000 ms, field of view = 30 × 32 mm, echo time = 38.64 ms, and slice thickness = 1 mm.

### Immunohistochemistry (IHC)

Paraffin-embedded sections were subjected to IHC. After deparaffinization and rehydration, sections were exposed to primary antibodies overnight, rinsed three times, and then incubated with appropriate species-specific secondary antibodies for 1 h at room temperature. Signals were developed with 3,3^’^-diaminobenzidine (DAB), and images were acquired using an Olympus microscope (Japan).

### RNA sequencing

For RNA sequencing, PANC-1 cells treated with or without dronedarone were collected in triplicate. RNA quantity and integrity were evaluated on an Agilent 2100 Bioanalyzer (Agilent Technologies, USA). Sequencing libraries were constructed using the NEBNext^®^ Ultra™ RNA Library Prep Kit for Illumina^®^ (NEB, USA) and sequenced on an Illumina NovaSeq 6000 platform (Illumina, USA) with 150 bp paired-end reads. Differentially expressed genes (DEGs) were defined as genes with an adjusted *P* value (FDR) < 0.05 and |log2(fold change) | > 1.5. Using the Kyoto Encyclopedia of Genes and Genomes (KEGG) database, pathway annotation and enrichment analysis were performed for the DEGs.

### Lipid peroxidation assay

Pancreatic cancer cells were treated with dronedarone, stained with BODIPY 581/591 C11 (Thermo Fisher Scientific, USA) for 1 h at 37 °C, rinsed with PBS, and imaged on a fluorescence microscope. The signals from reduced and oxidized C11-BODIPY were captured using a fluorescence microscope, with emission collected at 590 nm and 510 nm, respectively.

### GSH assay

To quantify intracellular GSH levels, white 96-well plates (#137101, Thermo Fisher Scientific, USA) were used for overnight cell seeding, followed by dronedarone treatment for the indicated times. In accordance with the manufacturer’s protocol, GSH levels were measured using the GSH/GSSG-Glo Assay (Promega, USA).

### Western blotting

Cell lysates were prepared in cell lysis buffer (Beyotime, China) supplemented with protease and phosphatase inhibitors. Equal amounts of protein were loaded onto SDS-PAGE gels and transferred to nitrocellulose membranes. Membranes were blocked and sequentially incubated with primary and corresponding HRP-conjugated secondary antibodies. Signals were developed using enhanced chemiluminescence (ECL) and visualized with a western blot imaging system (Jiapeng, China). Band intensities were quantified using ImageJ.

### Transmission electron microscopy (TEM)

Following dronedarone treatment, pancreatic cancer cells were fixed with EM fixative solution (Servicebio, China) at 4 °C overnight. After three washes were performed with phosphate buffer, samples were post-fixed with 1% osmic acid for 2.5 h at room temperature. Samples were dehydrated in graded ethanol, resin-embedded, ultrathin-sectioned, and contrasted with uranyl acetate and lead citrate before TEM imaging.

### Mitotracker staining

Mitochondria in live cells were stained with MitoTracker Red CMXRos (Invitrogen, USA). Probe staining was performed at 100 nM for 30 min at 37 °C, and nuclei were counterstained with Hoechst. Confocal images were captured using a laser-scanning microscope, and mitochondrial morphology was quantified in Fiji software.

### ROS detection

Intracellular ROS were detected using DCFH-DA (Biosharp, China). Cells were incubated with 10 µM DCFH-DA diluted in serum-free medium for 15 min at 37 °C in the dark, washed with PBS, and analyzed by fluorescence microscopy (IX73 + DP74, Olympus, Japan) and flow cytometry. Mitochondrial ROS were measured using 5 µM MitoSox Red staining (Thermo Fisher Scientific, USA) according to the manufacturer’s instructions.

### Mitochondrial membrane potential (MMP)

The JC-1 Mitochondrial Membrane Potential Assay Kit (Invitrogen, USA) was used to assess MMP. After treatment, cells were incubated with JC-1 working solution at 37 °C for 20 min and then rinsed with JC-1 buffer. Images were acquired using a fluorescence microscope. MMP was determined by comparing RFP and GFP fluorescence intensities.

### scRNA-seq and data preprocessing

Fresh tumor tissues from KPC mice were enzymatically dissociated with MobiSolution™ Tissue Dissociation Kit. Cell suspensions with > 80% viability were processed for scRNA-seq. Single-cell suspensions were adjusted to 700-1,200 cells/µL. The suspensions were then loaded onto chips, and droplets were generated using the MobiNova-100 system. Using the MobiDrop kit (PN-S050200301, China), reverse transcription, cDNA amplification, and library construction were performed following the manufacturer’s protocol. MobiVision software (version 3.2) was used to generate FASTQ files, which were aligned to the mouse reference genome (GRCm39) with unique molecular identifiers (UMIs) counted per barcode. Low-quality cells (gene number < 200, maximum genes > 90%, and mitochondrial genes > 10%) were filtered. In total, 58,129 cells were obtained for downstream bioinformatic analysis. The sequencing and data preprocessing were conducted by OE Biotech Co., Ltd. (China).

### Cell clustering analysis and annotation

Cell-clustering was performed using the Seurat FindClusters function. Two-dimensional visualization was generated by uniform manifold approximation and projection (UMAP).

Seurat package FindAllMarkers was used to identify cluster-specific markers with log2FC > 0.5 and min.pct > 0.25. Cell clusters were annotated based on highly expressed genes, uniquely expressed genes, and canonical markers reported in the literature. Gene signature scores for ferroptosis (KEGG mmu04216), autophagy (KEGG mmu04140), and oxidative stress response (GO:0006979) were calculated using Seurat AddModuleScore and UCell algorithms.

### Autophagy detection system

Autophagy flux was assessed using an mCherry-EGFP-LC3B plasmid (GenePharma, China). Pancreatic cancer cells were infected with the plasmid and cultured for 48 h with or without dronedarone. Cells were imaged using a 63x oil confocal laser-scanning microscope (SpinSR, Olympus, Japan). The results were then analyzed using Image J software.

### GPX4 activity assay

The GPX4 Activity Assay Kit (K883, Elabscience, China) was used to measure the activity of GPX4. After treatment, cells were lysed in extraction buffer and clarified by centrifugation. The test solution was added to the supernatants and OD340 was measured on a FlexStation 3 microplate reader.

### RNA interference and gene transfection

Short hairpin RNA (shRNA) was used to reduce GPX4 expression in PANC-1 cells. Scrambled shRNA (P72558) and GPX4-targeting shRNAs (P41197 and P41198) were designed by Miaolingbio (China). Transfections were performed as previously described. The sequences of shRNA are available at http://www.miaolingbio.com/. Small interfering RNA (siRNA) was used to silence p62 expression. The siRNA sequences were as follows: si-p62-1, 5’-GGAGTCGGATAACTGTTCA-3’; si-p62-2, 5’-TGAGGAAGATCGCCTTGGA-3’. The cDNAs of human wild-type (WT) GPX4 and its mutant forms were synthesized and cloned into pcDNA3.1 by GenePharma (China).

### Molecular docking

Structure data file (SDF) of dronedarone was retrieved from the PubChem, and the human GPX4 structure was downloaded from the Protein Data Bank (PDB). The receptor was prepared in PyMOL by deleting water molecules and co-crystallized ligands. Structure preparation in AutoDock Tools included the addition of polar hydrogens and assignment of partial charges, after which the structure was saved as a PDBQT file. Molecular docking was carried out using PyRx (AutoDock Vina) with GPX4 as the receptor and dronedarone as the ligand. Predicted binding energies (kcal/mol) and docking outputs were recorded, and the lowest-energy pose was retained. Complexes were visualized in PyMOL and Discovery Studio 2020 Client.

### Surface plasmon resonance (SPR)

Recombinant human GPX4 (U73S, Cusabio, China) protein was immobilized on a CM5 sensor chip (BR-1005-30, Cytiva, Sweden). Increasing concentrations of dronedarone were flowed over the chip at 30 µl / min for 150 s. Measurements were acquired on a Biacore 8 K (GE Healthcare, USA) instrument, and data were processed with Biacore Insight Evolution software (Cytiva, USA).

### Immunoprecipitation (IP)

After dronedarone treatment, IP lysis buffer (87788, Thermo Fisher, USA) was used to lyse cells. Lysates were incubated with the indicated antibody at 4 °C overnight, and immune complexes were then captured with Protein A/G agarose beads for 1 h. After collection and washing with lysis buffer, immune complexes were analyzed by SDS-PAGE.

### Immunofluorescence (IF) analysis

Cells were plated on confocal dishes for IF staining. After treatment, cells were fixed with 4% paraformaldehyde and permeabilized with 0.1% Triton X-100. After deparaffinization and rehydration, paraffin-embedded mouse tumor sections were processed for IF staining. Cells and tissue sections were incubated with blocking buffer for 1 h, followed by incubation with primary antibodies overnight at 4 °C. Fluorescent secondary antibodies were applied for 1 h. Fluorescence images were acquired using an Olympus confocal microscope (SpinSR, Japan).

### Statistical analysis

Data are presented as mean ± SEM and were analyzed with GraphPad Prism (GraphPad, USA). One-way ANOVA was used for multi-group comparisons. Two-group comparisons were made using a two-tailed Student’s *t*-test. Statistical significance was defined as *P* < 0.05, with significance levels indicated as follows: *P < 0.05, **P < 0.01, ***P < 0.001, and ****P < 0.0001.

## Results

### High-throughput screening (HTS) identifies dronedarone as a potent inducer of pancreatic cancer cell death

**Fig. 1:**
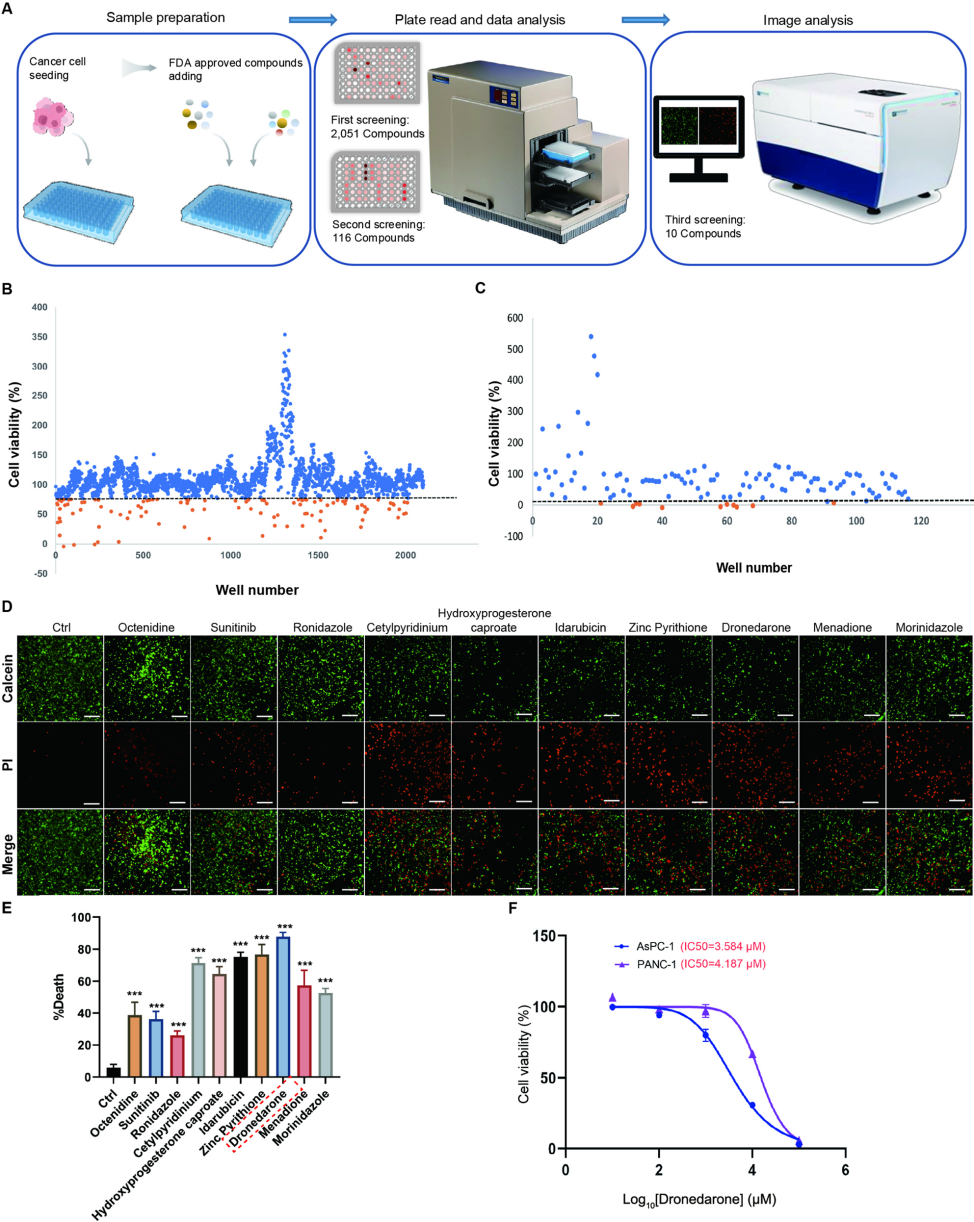
High-throughput screening identifies dronedarone as a potent inducer of pancreatic cell death. **A** Schematic diagram of the high-throughput screening platform using a library of 2,051 FDA-approved compounds. **B** Primary screen identifying compounds that inhibited PANC-1 cell viability after a 24 h drug exposure. Each dot represents one compound. A total of 116 drugs with cell viability < 75% (dashed line) passed the primary screen. **C** Secondary drug screen. Compounds reducing cell viability to < 10% were considered positive hits. **D** The top 10 candidate compounds were further validated using live/dead staining. **E** Quantification of live and dead cells after 24 h treatment at 10 µM. This assay was used to confirm cytotoxicity relative to the vehicle control. **F** Dose–response curves and calculated half-maximal inhibitory concentrations (IC50) of dronedarone in AsPC-1 and PANC-1 cells after 24 h treatment, determined by CCK-8 assay. Data are presented as mean ± SEM. ***P < 0.001

To identify agents that could induce cell death in pancreatic cancer, we established an HTS platform (Fig. 1A) using a library of 2,051 FDA-approved compounds (Supplementary Table 1). Each compound within this library was employed to treat PANC-1 cells, and cell growth inhibition ratio was quantified relative to controls (vehicle-treated). In the primary screen, a threshold was established whereby the post-treatment survival rate of PANC-1 cells had to be < 75%, resulting in the identification of 116 compounds (Fig. 1B). In the secondary screen, a threshold of < 10% cell viability was applied, leading to the identification of 10 compounds (Fig. 1C, Supplementary Table 2). In the third round of selection, a live/dead assay was used to directly quantify cell death, and dronedarone elicited significant cell death in PANC-1 cells (Fig. 1D-E). Notably, both dronedarone and dronedarone hydrochloride were included in the screening, however, only dronedarone met the secondary-screen hit threshold, whereas dronedarone hydrochloride did not (Fig. S1A). To further evaluate the ability of dronedarone to induce cell death, we examined its effect in AsPC-1 and PANC-1 cells. Dose-response curves showed that half-maximal inhibitory concentration (IC50 values) of dronedarone were 3.584 µM and 4.187 µM for AsPC-1 and PANC-1 cells, respectively (Fig. 1F). Collectively, these three rounds of screening identify dronedarone as a potent inducer of pancreatic cancer cell death.

### Anticancer activity of dronedarone in pancreatic cancer

**Fig. 2:**
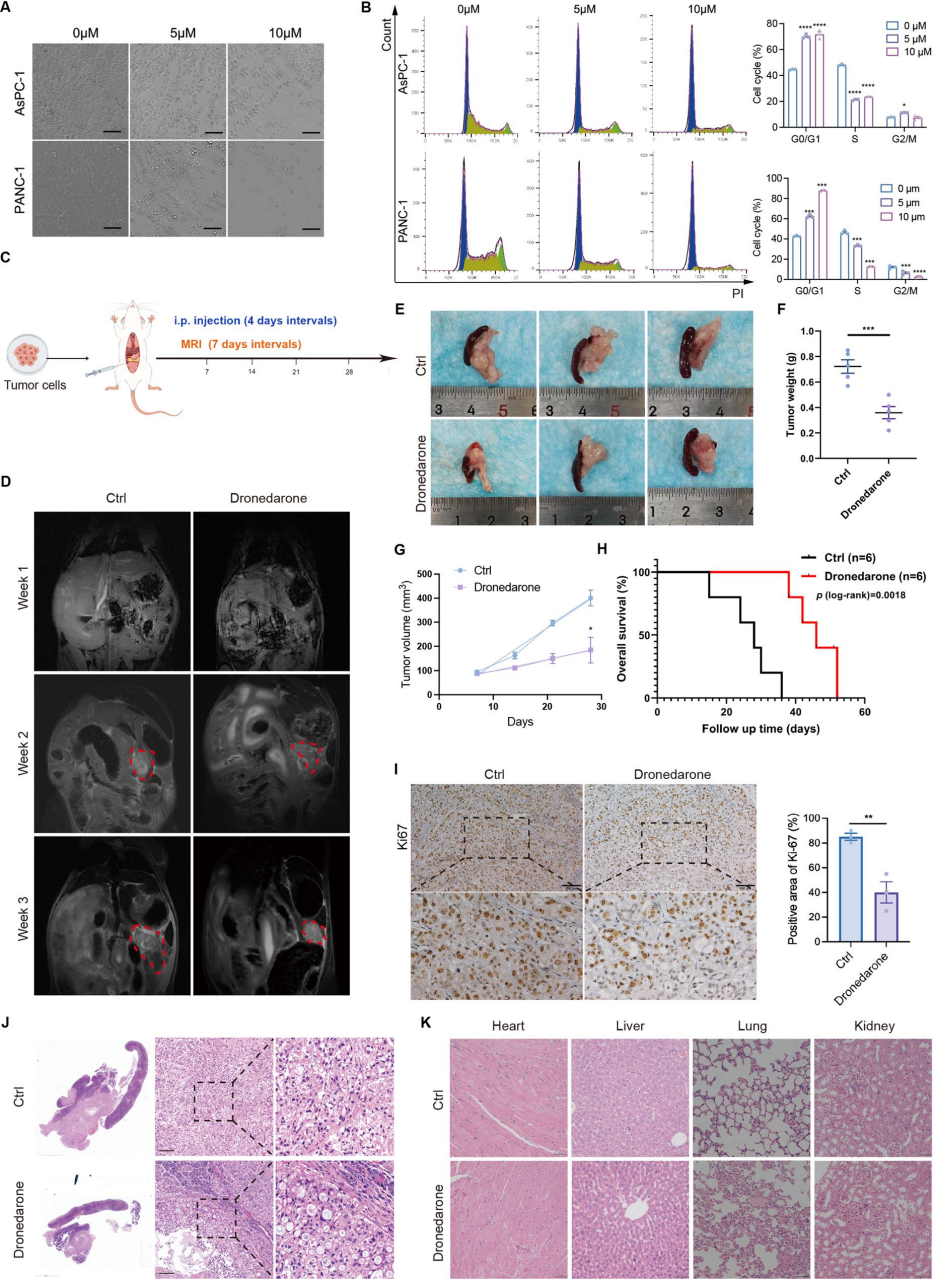
Anticancer activity of dronedarone in pancreatic cancer. **A** Representative bright field images of pancreatic cancer cells treated with vehicle or dronedarone for 24 h. **B** Effects of dronedarone on cell cycle of AsPC-1 and PANC-1 cells. **C** Schematic diagram of the in vivo experimental design. **D** Representative in vivo T2-weighted MRI images of mice treated with vehicle or dronedarone. **E** Representative photographs of tumors collected from mice at the study endpoint. **F** Tumor weights at the study endpoint. **G** Tumor growth curves of vehicle- and dronedarone-treated mice derived from MRI measurements. . **H** Survival curves. I Representative immunohistochemistry images of Ki67 staining. **J** Representative hematoxylin and eosin (HE) staining of tumors. **K** Representative HE staining of heart, liver, lung and kidney from vehicle- and dronedarone-treated mice. Data are presented as mean ± SEM. *P < 0.05, **P < 0.01, ***P < 0.001, and ****P < 0.0001

To assess antitumor activity, we exposed pancreatic cancer cells to 0, 5, or 10 µM dronedarone according to the IC50 values. After treatment for 24 h, cells were swollen, flattened and showed slower proliferation, and exhibited a significant decrease in cell number (Fig. 2A). Colony formation assays confirmed that dronedarone markedly reduced colony-forming ability, with almost no colonies observed at the higher concentrations (Fig. S1B-C). Consistently, we found that as the drug concentration increased, dronedarone markedly increased the proportion of cells in the G0/G1 phase (Fig. 2B), indicating that dronedarone effectively inhibits cell proliferation.

We next evaluated the antitumor activity of dronedarone in vivo (Fig. 2C). The results showed that dronedarone significantly slowed tumor growth in vivo compared with the control group (Fig. 2D). At the study endpoint, tumors from dronedarone-treated mice were substantially smaller and lighter than those from control mice (Fig. 2E-G). Dronedarone also prolonged survival, increasing median survival from 28 days in the control group to 46 days in the dronedarone-treated group (Fig. 2H). IHC of Ki67 showed a significant reduction in proliferating tumor cells (Fig. 2I). Hematoxylin and eosin (HE) staining revealed increased tumor cell death without obvious histopathologic damage in major organs (Fig. 2J-K). To evaluate the potential cardiotoxicity of dronedarone, we determined its IC50 in human cardiomyocyte AC16 cells. The 24 h IC50 value was 12.190 µM, higher than the IC50 values in pancreatic cancer cells, indicating a therapeutic window in which antitumor effects are achieved with limited cardiotoxicity (Fig. S1D). Collectively, these findings demonstrate that dronedarone exerts antitumor activity against pancreatic cancer both in vitro and in vivo.

### Dronedarone induces ferroptosis in pancreatic cancer cells

**Fig. 3:**
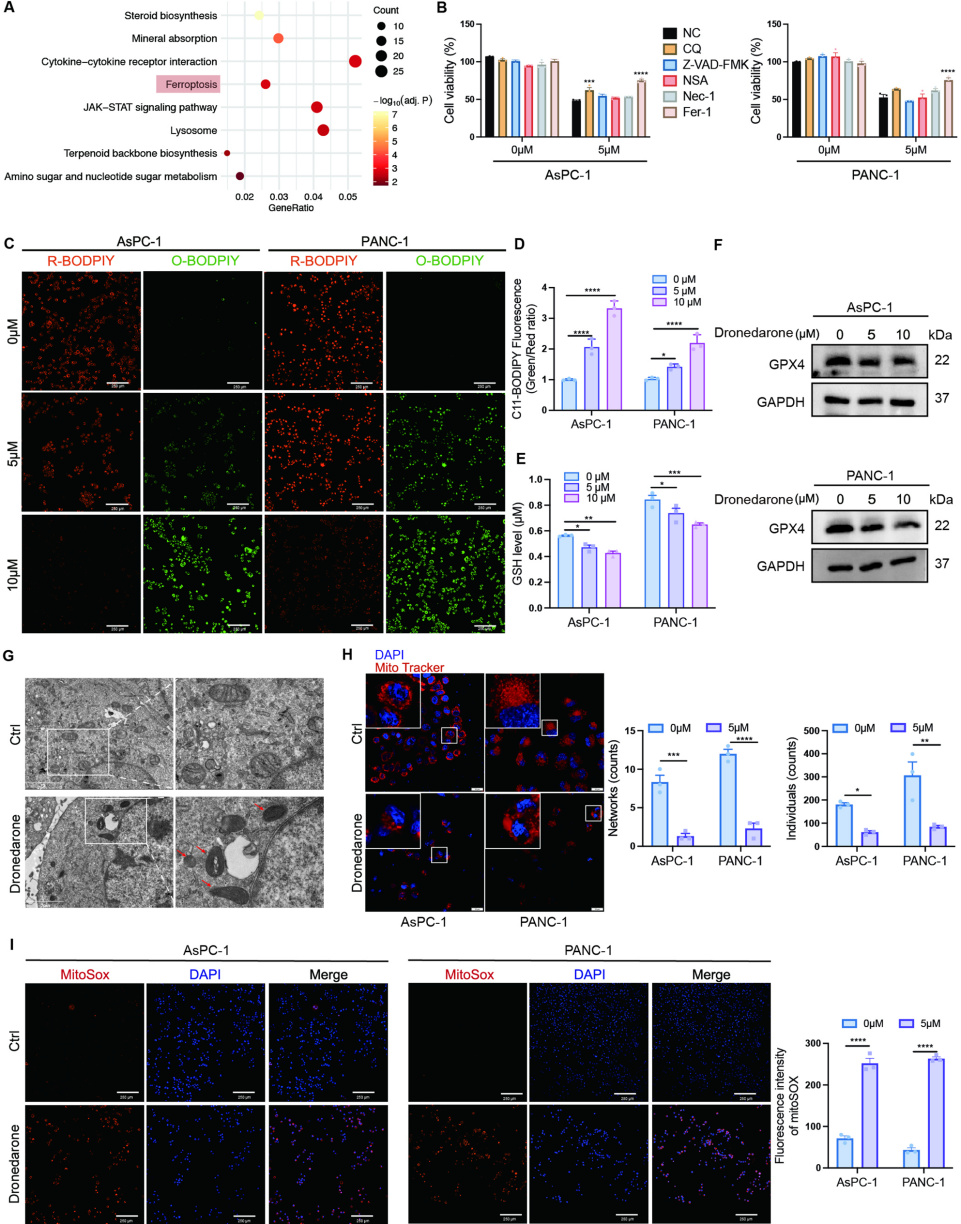
Dronedarone induces ferroptosis in pancreatic cancer cells. **A** The top 8 up enrichment KEGG pathways in dronedarone-treated group. **B** Cell viability of pancreatic cancer cells treated with dronedarone in the presence of inhibitors of apoptosis (Z-VAD-FMK), necrosis (necrostatin-1), necroptosis (necrosulfonamide), autophagy (chloroquine), or ferroptosis (ferrostatin-1). **C** Representative images of BODIPY 581/591 C11 staining. **D** Quantitation of the lipid peroxidation ratio (green/red) normalized to the control group. **E** Cellular glutathione levels in pancreatic cancer cells treated with vehicle or dronedarone. Statistics were performed within each cell line (vehicle vs. dronedarone). **F** Representative western blots of GPX4 protein expression in pancreatic cancer cells treated with vehicle or dronedarone. **G** Transmission electron microscopy images of mitochondrial ultrastructure in pancreatic cancer cells. **H** Representative images of MitoTracker-labeled mitochondria in AsPC-1 and PANC-1 cells treated with vehicle or dronedarone, together with quantification of the mitochondrial morphology. **I** MitoSOX staining of mitochondrial superoxide in vehicle- and dronedarone-treated pancreatic cancer cells. Data are presented as mean ± SEM. *P < 0.05, **P < 0.01, ***P < 0.001, and ****P < 0.0001

To elucidate the underlying mechanism, we performed RNA-seq. Compared with the control group, there were 1517 genes upregulated and 1187 genes downregulated in the dronedarone group (Fig. S2A). KEGG pathway analysis highlighted ferroptosis as the most significantly enriched pathway (Fig. 3A). Given this, we performed pharmacological rescue assays using inhibitors of apoptosis, necrosis, necroptosis, autophagy, and ferroptosis. Among these agents, Fer-1, a ferroptosis inhibitor, most effectively reversed dronedarone-induced loss of viability (Fig. 3B). In addition, we evaluated the effect of the pyroptosis inhibitor DSF in a co-treatment assay (Fig. S2B).

Ferroptosis is driven by an imbalance between oxidant and antioxidant defenses culminating in ROS-mediated lipid peroxidation [15]. Using BODIPY 581/591 C11, we observed a marked increase in lipid ROS following dronedarone treatment (Fig. 3C-D). Consistently, dronedarone increased ROS (Fig. S2C) and decreased reduced GSH (Fig. 3E). Given the central role of the cystine/glutamate antiporter system Xc- and its downstream effector GPX4 in limiting lipid peroxidation, we assessed GPX4 expression and found that dronedarone reduced GPX4 protein levels in pancreatic cancer cells (Fig. 3F). Densitometric quantification normalized to GAPDH confirmed a significant reduction (Fig. S2D).

Ferroptosis is typically accompanied by mitochondrial structural and functional abnormalities. TEM revealed that dronedarone induced mitochondrial shrinkage, cristae loss, and increased membrane density, without chromatin condensation or nuclear fragmentation-features consistent with ferroptosis (Fig. 3G). Confocal imaging of MitoTracker-labeled cells further showed that, after dronedarone treatment, the mitochondrial network was disrupted and highly fragmented (Fig. 3H). JC-1 staining assays revealed a decreased red/green fluorescence ratio, reflecting a loss of MMP after dronedarone intervention (Fig. S2E). MitoSOX staining revealed an increase in mitochondrial superoxide levels in pancreatic cancer cells after dronedarone treatment (Fig. 3I). Collectively, our findings demonstrate that dronedarone induces ferroptosis in pancreatic cancer cells.

### scRNA-seq profiling of KPC tumors treated with dronedarone

**Fig. 4:**
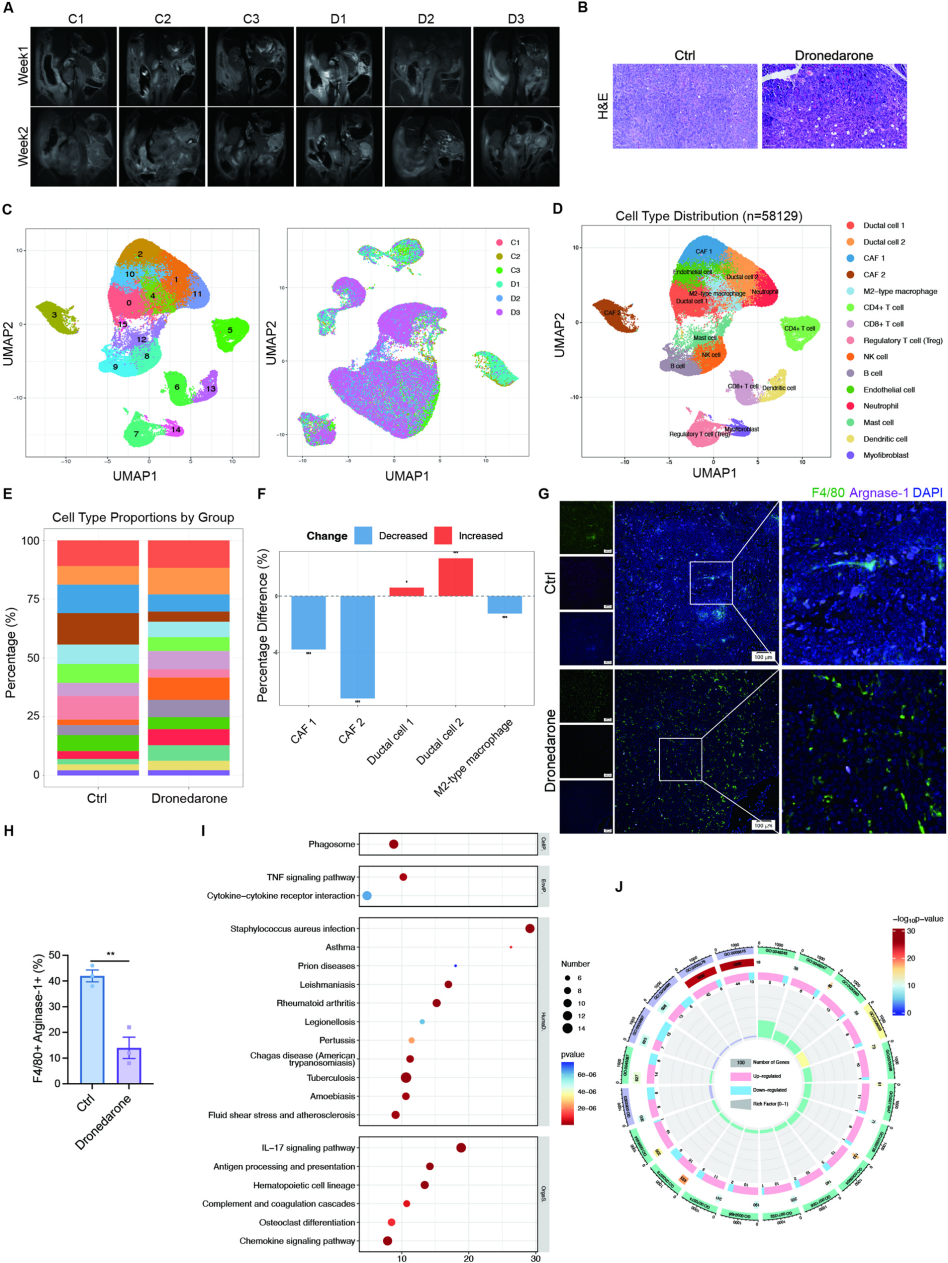
Single-cell RNA-seq profiling of KPC tumors treated with dronedarone. **A** Representative MRI images of KPC mice treated with vehicle or dronedarone. **B** HE staining of KPC tumors. **C** Uniform manifold approximation and projection (UMAP) visualization of 58,129 single cells from vehicle- and dronedarone-treated KPC tumors. **D** UMAP plot with clusters colored by cell type. **E** Cell type proportions in vehicle- and dronedarone-treated groups. **F** Cell-type-specific differential expression analysis of cancer-associated fibroblasts (CAFs), M2-type macrophages and pancreatic ductal cells in vehicle- and dronedarone-treated tumors. **G** Representative immunofluorescence images of F4/80 and arginase-1 staining in KPC tumor sections from vehicle- and dronedarone-treated mice. **H** Quantification of M2-type macrophages in tumors. **I** KEGG pathway analysis of differentially expressed genes (DEGs) between vehicle- and dronedarone-treated tumors. **J** GO enrichment analysis. Data are presented as mean ± SEM. *P < 0.05, **P < 0.01, ***P < 0.001, and ****P < 0.0001

To further understand the molecular pathways associated with ferroptosis following dronedarone treatment, we performed scRNA-seq on tumors from KPC mice treated with vehicle or dronedarone. Tumor formation in KPC mice was monitored by MRI (Fig. 4A) and histologically confirmed as pancreatic cancer by HE staining (Fig. 4B). After quality control, 58,129 cells were retained for subsequent analysis. Cellular composition was assessed by unbiased clustering across all cells using principal component analysis (PCA) and visualized using UMAP. We identified 16 clusters representing different cell types. All the samples showed similar overall cell-type compositions and proportions (Fig. 4C). The top 10 markers expressed in each cluster were displayed with a heatmap (Fig. S3A). Then we annotated clusters using lineage markers and identified 15 major cell types (Fig. 4D). Cluster 15 was not defined due to the lack of known marker gene expression and functional pathways and was excluded from further analysis. Characteristic genes for each cell cluster are presented in a dot plot (Fig. S3B). The percentages of all cell types within each group are shown in Fig. 4E.

Tumor cells exhibited two distinct populations, denoted as pancreatic ductal cell 1 and 2. The most pronounced compositional changes following dronedarone treatment were observed in ductal cells, cancer-associated fibroblasts (CAFs), and M2-type macrophages (Fig. 4F). Moreover, IF staining for F4/80 and Arginase-1 in tumor tissues of KPC mice showed a reduction in M2-type macrophages in dronedarone-treated mice (Fig. 4G-H). We next performed DEGs analysis between vehicle- and dronedarone-treated tumors (Fig. S3C), followed by Gene Ontology (GO) and KEGG enrichment analysis. These analyses indicated that genes differentially expressed in response to dronedarone were significantly enriched for lysosome- and phagosome-related pathways (Fig. 4I-J).

### Dronedarone enhances autophagic flux in pancreatic cancer cells

**Fig. 5:**
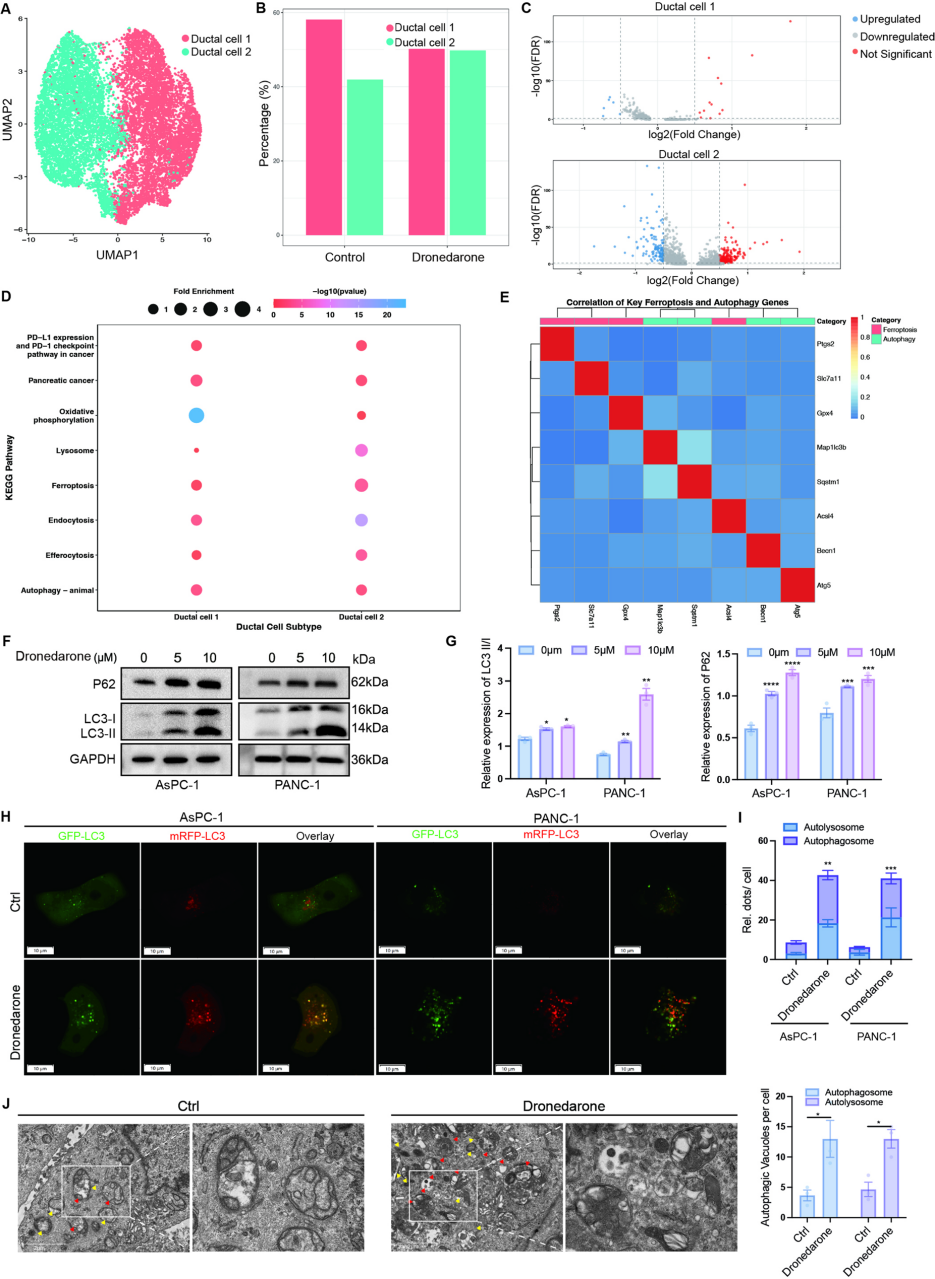
Dronedarone enhances autophagic flux in pancreatic cancer cells. **A** UMAP visualization of ductal cell 1 and 2 subtypes isolated from scRNA-seq data of vehicle- and dronedarone-treated KPC tumors. **B** Proportions of ductal cell 1 and 2 subtypes in vehicle- and dronedarone-treated groups. **C** Differentially expressed genes (DEGs) identified within ductal cell 1 and 2 subtypes. **D** KEGG pathway enrichment analysis of DEGs in ductal cell 1 and 2. **E** Correlation analysis of ferroptosis- and autophagy-related marker genes in ductal cell clusters. **F** Western blot analysis of LC3 and p62 protein expression in pancreatic cancer cells treated with increasing concentrations of dronedarone. **G** Quantification of LC3 and p62 protein expression. **H** Representative confocal images of mCherry-EGFP-LC3 reporter system-labeled autophagosomes (yellow puncta) and autolysosomes (red puncta) in PANC-1 and AsPC-1 cells treated with vehicle or dronedarone. **I** Quantification of autophagosomes and autolysosom**e**s per cell based on mCherry-EGFP-LC3 reporter system. **J** Autophagosomes and autophagy-lysosomes in pancreatic cancer cells under transmission electron microscopy. Data are presented as mean ± SEM. *P < 0.05, **P < 0.01, ***P < 0.001, and ****P < 0.0001

Notably, ductal cells were more abundant in dronedarone-treated tumors than in vehicle-treated tumors. To identify the cells that play a crucial role with their expression characteristics, we focused on the scRNA-seq data of ductal cells. The ductal cells exhibited remarkable heterogeneity (Fig. S4A). Dronedarone treatment reduced the proportion of the ductal cell 1 subtype while increasing the proportion of the ductal cell 2 subtype compared with controls (Fig. 5A-B). We then extracted genes from the scRNA-seq dataset of ductal cell 1 and 2 subtypes and performed differential gene expression analysis. A total of 309 DEGs were identified in ductal cell 1, and 637 DEGs were identified in ductal cell 2 (Fig. 5C). GO enrichment analysis of signature genes in each ductal subcluster showed distinct functional enrichment, including RNA splicing, protein localization to cell surface, and ribonucleoprotein complex biogenesis (Fig. S4B). Furthermore, KEGG pathway analysis demonstrated that DEGs were significantly enriched in terms related to pancreatic cancer, ferroptosis, oxidative phosphorylation and autophagy (Fig. 5D), indicating a major role for ferroptosis in dronedarone-induced tumor cell death. We noticed that autophagy was the only term commonly enriched in ductal cell 1 and 2 subtypes, suggesting that autophagy may contribute to the regulation of ferroptosis. Further correlation analysis revealed that GPX4 was substantially associated with Map1lc3b (LC3) and Sqstm1 (p62) (Fig. 5E), providing evidence that autophagy plays a role in dronedarone-induced ferroptosis. Moreover, we calculated ferroptosis, autophagy, and oxidative stress gene signature scores across all annotated cell types. The enrichment patterns were largely consistent across scoring methods (Fig. S5A-B). Therefore, these findings primarily support pathway-level activation associated with treatment.

During autophagosome biogenesis, cytosolic LC3 (LC3-I) is lipidated to form membrane-bound LC3-II, which is ultimately degraded in autolysosomes along with the autophagy receptor p62 [16]. Accordingly, the occurrence of autophagy is typically associated with reduced p62 levels. To determine the effect of dronedarone on autophagy, we performed western blotting for LC3 and p62. Unexpectedly, dronedarone induced a dose-dependent increase in p62 abundance (Fig. 5F-G). This pattern is compatible with ferroptosis-related stress, which can enhance autophagic activity while transcriptionally inducing p62 accumulation. We used the mCherry-EGFP-LC3 reporter system to quantify autophagic flux, counting yellow signals (mCherry and EGFP co-localization, autophagosomes) and red signals (mCherry only, autolysosomes) by confocal microscopy (Fig. 5H-I). Dronedarone increased both autophagosomes and autolysosomes, with a higher proportion of red signals, indicating enhanced autophagic flux in PANC-1 and AsPC-1 cells. TEM corroborated these findings, revealing numerous double-layered membrane autophagic vesicles in dronedarone-treated cells compared with controls (Fig. 5J). Taken together, these data indicate that dronedarone enhances autophagic flux in pancreatic cancer cells.

### Autophagy promotes dronedarone-induced ferroptosis in pancreatic cancer cells

**Fig. 6:**
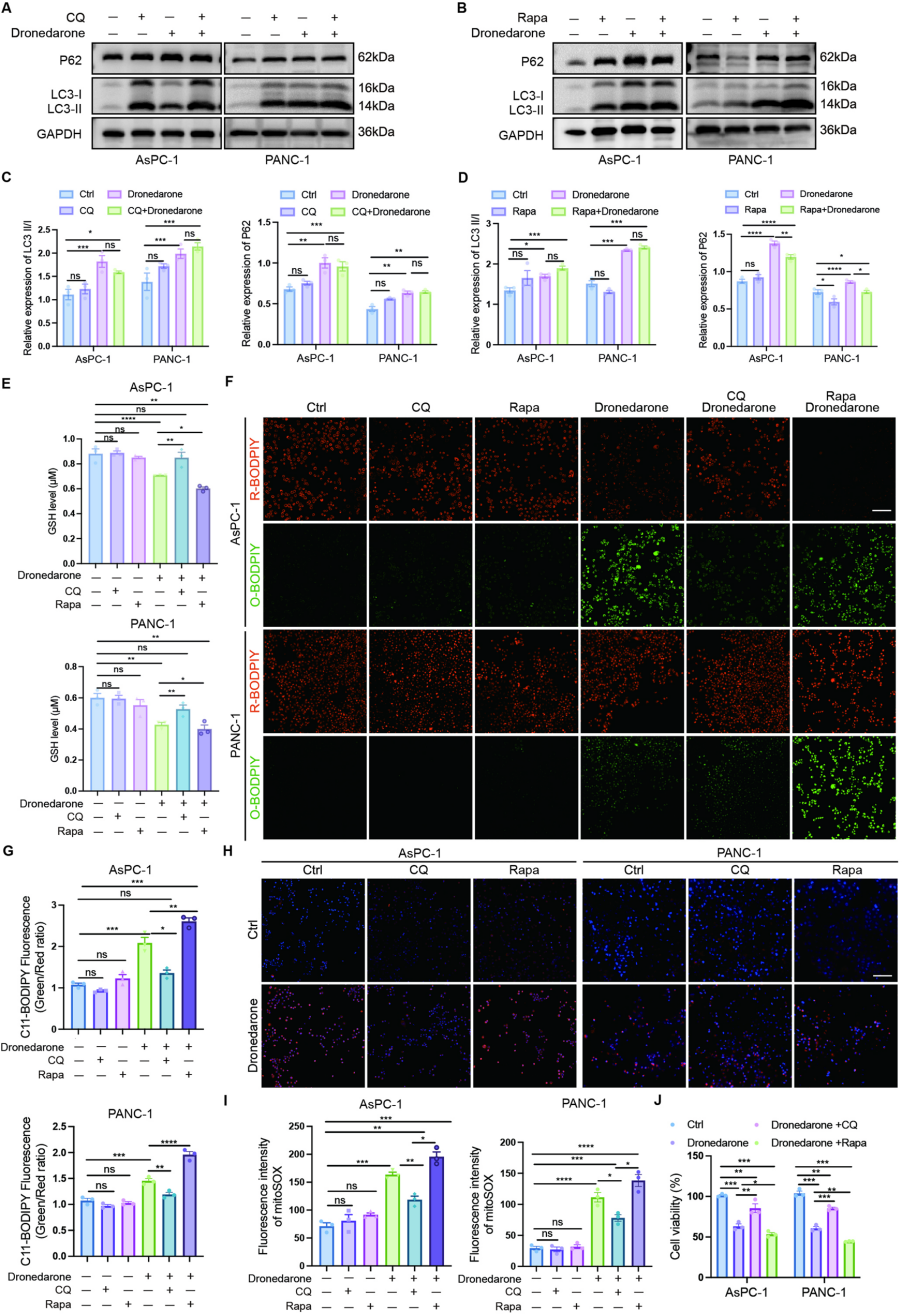
Autophagy modulates dronedarone-induced ferroptosis in pancreatic cancer cells. **A** Representative western blot analysis of LC3 and p62 protein levels in pancreatic cancer cells treated with vehicle, dronedarone, chloroquine (CQ), or the combination of dronedarone and CQ. **B** Representative western blot analysis of LC3 and p62 protein levels in pancreatic cancer cells treated with vehicle, dronedarone, rapamycin (Rapa), or the combination of dronedarone and Rapa. **C** Quantification of LC3-II/LC3-I ratio and p62 protein levels in cells treated with vehicle, dronedarone, CQ, or the combination of dronedarone and CQ. **D** Quantification of LC3-II/LC3-I ratio and p62 protein levels in cells treated with vehicle, dronedarone, Rapa, or the combination of dronedarone and Rapa. **E** Intracellular glutathione levels in pancreatic cancer cells treated with dronedarone in presence or absence of CQ or Rapa. **F** Representative BODIPY-C11 fluorescence images showing lipid peroxidation in pancreatic cancer cells. **G** Quantification of BODIPY-C11 fluorescence intensity. **H** Representative images of mitochondrial ROS levels in pancreatic cancer cells treated with dronedarone alone or in combination with CQ or Rapa. **I** Quantification of mitochondrial ROS levels in the indicated treatment groups. **J** CCK-8 assay of cell viability in pancreatic cancer cells treated with dronedarone alone or in combination with CQ or Rapa. Data are presented as mean ± SEM. *P < 0.05, **P < 0.01, ***P < 0.001, and ****P < 0.0001

To further define the crosstalk between autophagy and ferroptosis, we used the autophagy inhibitor CQ and the autophagy inducers Rapa (Fig. 6A-B). CQ blocks autophagosome-lysosome fusion, thereby inhibiting late-stage autophagy. We found that CQ further increased dronedarone-induced LC3-II accumulation, whereas p62 levels remained unchanged (Fig. 6C), consistent with ferroptosis-associated stress transcriptionally inducing p62 despite impaired turnover. Rapa activated autophagy, as evidenced by an increased LC3B-II/I ratio and reduced p62, and further augmented dronedarone’s autophagy-inducing effect (Fig. 6D). We then assessed dronedarone-induced ferroptosis under conditions of autophagy inhibition or activation. CQ mitigated dronedarone-inducing GSH depletion. Conversely, Rapa exacerbated GSH depletion (Fig. 6E). BODIPY-C11 assays showed that CQ reversed dronedarone-induced lipid peroxidation, whereas Rapa produced the opposite effect (Fig. 6F–G). Additionally, the mitochondrial ROS overloading (Fig. 6H-I) and the decrease in mitochondrial membrane potential (Fig. S5C) caused by dronedarone were alleviated after CQ treatment, while Rapa further aggravated this response. CCK-8 assays indicated that CQ partially rescued cell viability from dronedarone exposure (Fig. 6J). Collectively, these data demonstrate that autophagy promotes dronedarone-induced ferroptosis in pancreatic cancer.

### Dronedarone promotes GPX4 degradation through the autophagy-lysosomal pathway

**Fig. 7:**
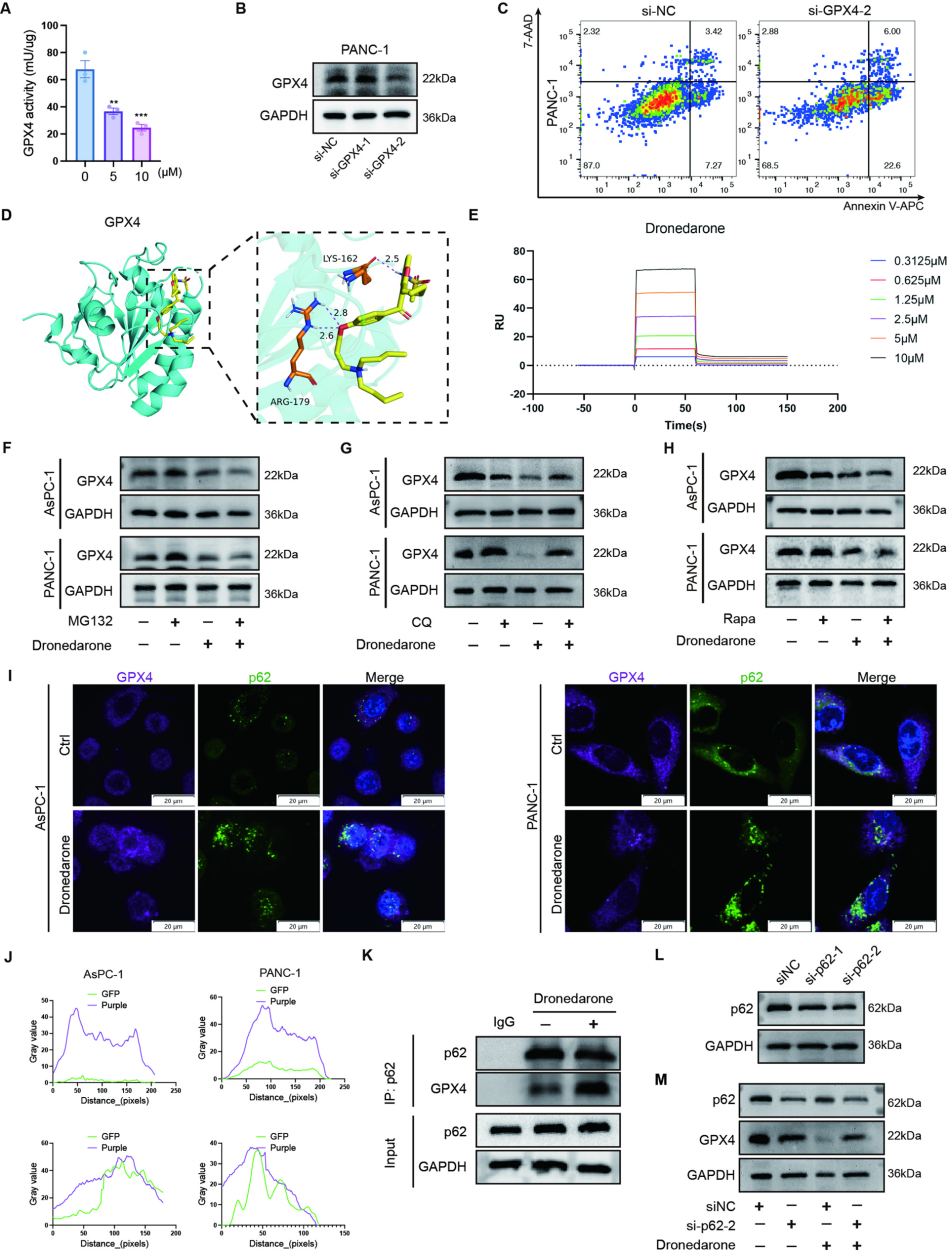
Dronedarone promotes GPX4 inactivation and degradation via the autophagy-lysosome pathway. **A** GPX4 enzymatic activity in PANC-1 cells treated with vehicle or dronedarone. **B** Western blot analysis confirming GPX4 knockdown efficiency in PANC-1 cells. **C** Flow cytometric analysis of apoptosis in control and GPX4-knockdown pancreatic cancer cells treated with dronedarone. **D** Molecular docking diagram of GPX4 and dronedarone. **E** Surface plasmon resonance (SPR) analysis of interaction between dronedarone and GPX4. (F-H) Western blot analysis of GPX4 protein levels in AsPC-1 and PANC-1 cells pre-treated with MG132 (**F**), CQ (G), or Rapa (**H**) followed by dronedarone treatment. **I** Representative confocal images showing co-localization of GPX4 (purple) and p62 (green). Scale bar = 20 μm. **J** Quantification of GPX4 and p62 co-localization signals in pancreatic cancer cells. **K** Co-immunoprecipitation (Co-IP) analysis of the interaction between GPX4 and p62 in PANC-1 cells treated with vehicle or dronedarone. **L** Representative western blots showing siRNA-mediated depletion of p62/SQSTM1 in PANC-1 cells. **M** Western blot analysis of GPX4 and p62 protein expression in PANC-1 cells transfected with si-NC or si-p62#2 and treated with dronedarone. Data are presented as mean ± SEM. *P < 0.05, **P < 0.01, ***P < 0.001, and ****P < 0.0001

We found that GPX4 was significantly downregulated in dronedarone-treated cells. Moreover, dronedarone inhibited GPX4 enzyme activity (Fig. 7A), indicating that dronedarone may bind directly to GPX4. As anticipated, GPX4 knockdown sensitized cells to death (Fig. 7B-C). Molecular docking predicted stable hydrogen bonds between GPX4 and dronedarone, highlighting K162 and R179 as key interaction residues (Fig. 7D). To further confirm the affinity between dronedarone and GPX4, we performed SPR, which demonstrated direct binding of dronedarone to GPX4 with a KD value of 4.72 µM (Fig. 7E). We next assessed the functional relevance of these residues by transfecting cells with GPX4-WT or the corresponding alanine-substitution mutants (K162A and R179A). Western blotting confirmed comparable expression levels across constructs (Fig. S6A). Notably, basal GPX4 activity was significantly reduced by R179A but not by K162A, compared with GPX4-WT (Fig. S6B).

We then performed rescue assays and tested ferroptosis-related indicators. Expression of GPX4-WT improved cell viability, mitigated GSH depletion, and attenuated lipid peroxidation upon dronedarone exposure (Fig. S6C–F). Interestingly, the K162A mutant conferred stronger attenuation of dronedarone-triggered ferroptosis than GPX4-WT, whereas R179A exhibited reduced baseline viability and elevated basal oxidative status without diminishing the normalized response to dronedarone (Fig. S6C–F). Together, these findings suggest that GPX4 is a key functional regulator in dronedarone-triggered ferroptosis, with K162 being relevant to dronedarone responsiveness, while R179 mainly supports basal GPX4 catalytic function.

Next, we investigated how dronedarone triggered GPX4 protein degradation. Post-transcriptional protein degradation commonly proceeds via the ubiquitin-dependent proteasome or autophagy-lysosomal pathways. To pharmacologically block these pathways, we used the proteasome inhibitor MG132 or lysosomal inhibitor CQ. Western blot experiments showed that CQ, but not MG132, reversed the degradation of GPX4 induced by dronedarone (Fig. 7F-G). After Rapa treatment, the expression of GPX4 further decreased (Fig. 7H), suggesting that the autophagy-lysosome pathway is involved in GPX4 degradation (Fig. S6G-I). To further substantiate this mechanism, we performed immunofluorescence co-localization and co-IP. Confocal fluorescence imaging showed co-localization between GPX4 and p62 upon dronedarone treatment (Fig. 7J). Co-IP revealed minimal basal p62-GPX4 interaction, which was markedly enhanced by dronedarone (Fig. 7K). To provide genetic evidence, we used siRNA to deplete p62 in PANC-1 cells (Fig. 7L). Efficient knockdown of p62 was confirmed by western blotting (Fig. S6J). Consistently, p62 knockdown substantially attenuated the dronedarone-induced GPX4 protein degradation (Fig. 7M; Fig. S6K). Collectively, dronedarone promotes GPX4 inactivation and autophagy-dependent degradation, thereby driving ferroptosis in pancreatic cancer cells.

### Dronedarone’s antitumor effect is largely ferroptosis-dependent in pancreatic cancer

**Fig. 8:**
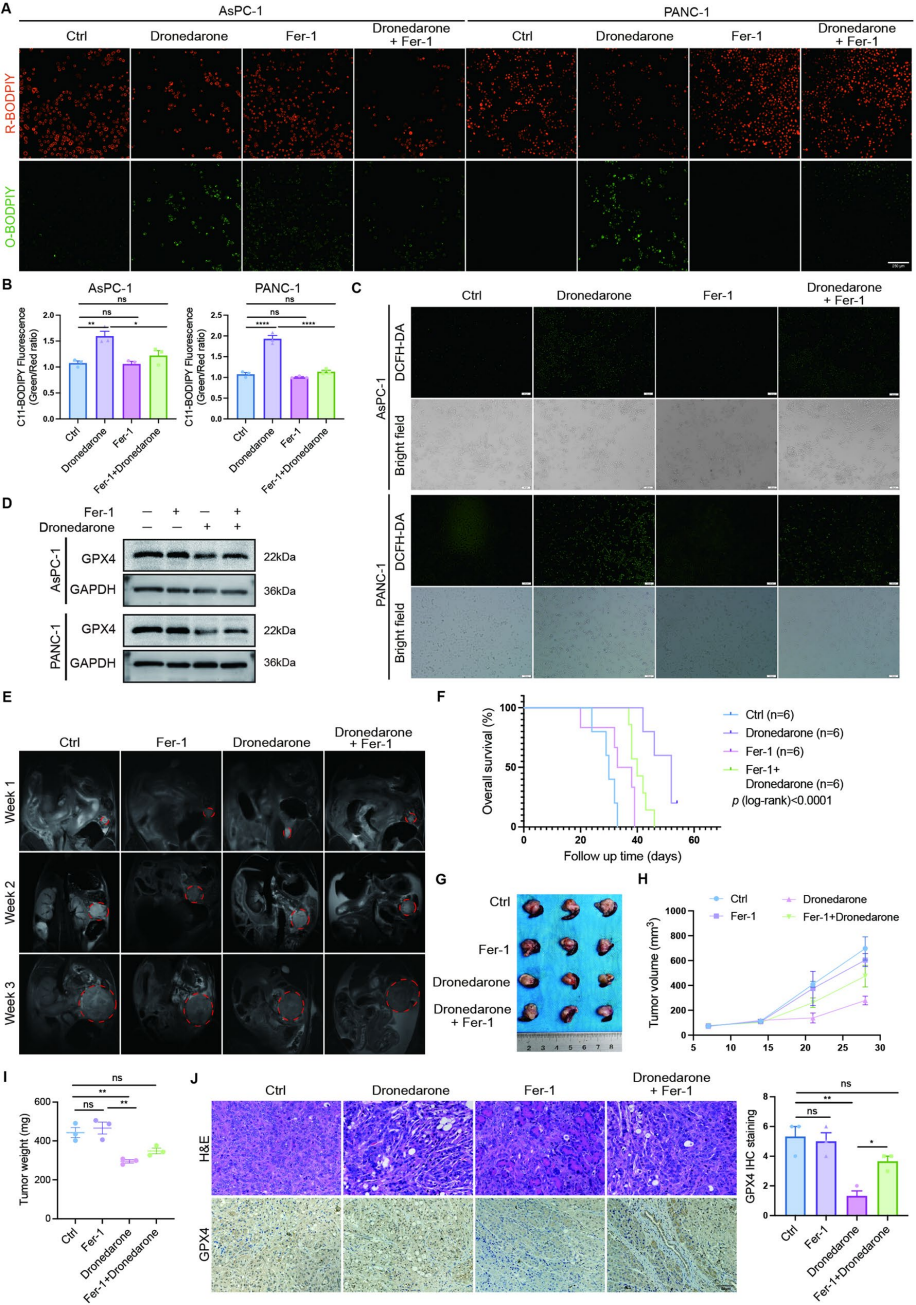
Dronedarone’s antitumor effect is largely ferroptosis-dependent in pancreatic cancer. **A** Representative fluorescence images of BODIPY 581/591 C11 staining. **B** Quantitation of the lipid peroxidation ratio (green/red) normalized to the control group. **C** Total ROS levels in pancreatic cancer cells. **D** Western blot analysis of GPX4 protein expression. **E** Representative MRI images of KPC mice treated with dronedarone in the presence or absence of Fer-1. **F** Kaplan-Meier survival curves of KPC mice. **G** Representative photographs of tumors collected from mice at the study endpoint. **H** Tumor growth curves of KPC mice. **I** Tumor weights at the study endpoint in the different treatment groups. **J** Representative hematoxylin and eosin (HE) staining of tumors, and immunohistochemistry images of GPX4 staining. Data are presented as mean ± SEM. For comparisons among four groups, statistical significance was determined by one-way ANOVA followed by Tukey’s multiple comparisons test. Survival analysis was performed using the log-rank (Mantel-Cox) test. *P < 0.05, **P < 0.01, ***P < 0.001, and ****P < 0.0001

To test whether ferroptosis is required for the antitumor activity of dronedarone, we used the ferroptosis inhibitor Fer-1. Fer-1 markedly reduced dronedarone-induced oxidation of BODIPY 581/591 C11, thereby lowering lipid peroxidation (Fig. 8A–B), and decreased total ROS following dronedarone treatment (Fig. 8C). By immunoblotting, Fer-1 preserved GPX4 abundance compared with dronedarone treatment alone (Fig. 8D; Fig. S7A). Serial monitoring demonstrated that dronedarone treatment markedly reduced tumor burden compared with vehicle, whereas co-treatment with Fer-1 partially reversed the effect (Fig. 8E). Kaplan-Meier survival analysis showed that median survival increased from 30 days in the vehicle group to 52 days with dronedarone, whereas Fer-1 exhibited only 35.5 days, and the combination reduced the survival benefit of dronedarone to 40 days (Fig. 8F). At the experimental endpoint, co-treatment with Fer-1 attenuated the antitumor effect of dronedarone, as reflected by increased tumor size and weight (Fig. 8G-I). Consistently, IHC showed reduced GPX4 expression after dronedarone monotherapy, whereas GPX4 level was maintained in the Fer-1 combination group (Fig. 8J). Consistent with the scRNA-seq findings, IHC confirmed that dronedarone reduced PD-L1 expression in tumor tissues by IHC (Fig. S7B). Furthermore, no obvious body weight changes (Fig. S7C) and organ damage were observed in any of the treated groups (Fig. S7D). Together, pharmacologic ferroptosis blockade reverses dronedarone-induced lipid peroxidation and tumor control, indicating that the antitumor activity of dronedarone is largely ferroptosis-dependent in pancreatic cancer.

## Discussion

Despite recent advances in systemic therapies for pancreatic cancer, clinical outcomes remain dismal. Targeting ferroptosis has emerged as a promising therapeutic strategy to overcome resistance to conventional apoptosis-based treatments [17]. In this study, we identified dronedarone, an FDA-approved antiarrhythmic agent, as a previously unrecognized promoter of ferroptosis in pancreatic cancer by performing a HTS of an FDA-approved compound library. Our findings showed that dronedarone exerts antitumor effects in part by directly binding to GPX4, thereby inactivating its enzymatic activity and promoting its degradation through the autophagy-lysosome pathway. These results expand the repertoire of ferroptosis-oriented agents and highlight a clinically accessible strategy for engaging ferroptosis vulnerability in pancreatic cancer.

Dronedarone is a benzofuran derivative of amiodarone that has been approved for the treatment of atrial fibrillation and atrial flutter, primarily through blockade of potassium, sodium and L-type calcium channels [18]. Large clinical trials have shown that dronedarone reduces atrial fibrillation recurrence and cardiovascular burden compared with other antiarrhythmic agents, and its pharmacokinetics and safety profile are well characterized [19, 20]. Beyond its antiarrhythmic indications, several studies have begun to explore the anticancer properties of dronedarone. In gastric cancer, dronedarone was reported to inhibit SRC kinase activity and suppress AKT1 phosphorylation, thereby restraining tumor growth [13]. In esophageal squamous cell carcinoma, dronedarone suppressed tumor proliferation in vitro and in vivo by directly binding to CDK4 and CDK6, reducing RB phosphorylation and inducing G1 cell-cycle arrest [21]. Specifically, therapy-induced cell cycle arrest has been reported to bidirectionally modulate ferroptosis susceptibility in a context-dependent manner, including cellular responses to GPX4 inhibition [22, 23]. For example, CDC26-mediated cycle block was shown to facilitate ferroptosis by promoting SLC7A11 degradation [24]. In contrast, cell cycle arrest has also been reported to induce lipid droplet formation, which can confer resistance to ferroptosis [25]. In our study, dronedarone inhibited pancreatic cancer growth and induced G1-phase arrest in vitro. However, whether dronedarone-induced ferroptosis is mechanistically linked to its effect on cell cycle suppression remains unclear. The molecular basis of dronedarone-induced G1 arrest in pancreatic cancer (e.g., CDK4/CDK6) warrants further investigation. Importantly, our data extend previous work by showing that, in addition to its effects on cell cycle, dronedarone promotes ferroptosis and exhibits a favorable safety profile in mice, in contrast to many standard pancreatic cancer drugs that are associated with severe toxicity [26–28]. RNA sequencing further supported that dronedarone-induced tumor cell death is a dominant biological event within the tumor ecosystem, making it an attractive candidate for repurposing in pancreatic cancer.

Our scRNA-seq of control and dronedarone-treated KPC tumors revealed that malignant cell clusters from the treated group were enriched for pathways related to ferroptosis and autophagy. Consistent with the central role of GPX4 in ferroptosis [29, 30], dronedarone markedly decreased GPX4 protein levels and was accompanied by mitochondrial dysfunction, accumulation of ROS and enhanced lipid peroxidation. Moreover, dronedarone-induced cytotoxicity was largely reversed by the ferroptosis inhibitor Fer-1 [31] both in vitro and in the KPC mouse model, supporting that ferroptosis represents a major component of the cell death program induced by dronedarone in pancreatic cancer. Consistently, overexpression of GPX4 partially protected pancreatic cancer cells from dronedarone-induced cytotoxicity, GSH depletion and lipid peroxidation, providing genetic evidence that GPX4 plays a functional role in dronedarone-triggered ferroptosis stress. Taken together, these data support that ferroptosis is the predominant mode of regulated cell death induced by dronedarone in pancreatic cancer, mediated in part through GPX4 degradation and excessive oxidative lipid damage. Given the pleiotropic pharmacology of dronedarone, additional targets and pathways may also contribute to the observed phenotype in a context-dependent manner.

The use of pharmacological modulators, including the autophagy agonists Rapa and inhibitors, helped to clarify how dronedarone promotes GPX4 degradation. Post-transcriptional protein degradation is predominantly mediated by the ubiquitin–proteasome system or the autophagy-dependent lysosomal pathway [32]. Notably, dronedarone-induced reduction of GPX4 protein levels and cell death were substantially reversed by the autophagy inhibitor CQ, but not by the proteasome inhibitor MG132, suggesting that GPX4 is preferentially degraded via the autophagy–lysosome pathway. Consistently, dronedarone treatment led to a marked increase in autophagosome and autolysosome formation, as monitored by the mCherry-EGFP-LC3 reporter, demonstrating activation of autophagic flux in pancreatic cancer cells.

Autophagy is a key homeostatic process that clears damaged proteins, organelles and invading pathogens through the lysosomal pathway, and its activation in cancer can exert either tumor-promoting or tumor-suppressive effects depending on context [16]. Selective autophagy relies on cargo receptors such as p62/SQSTM1, which recognize specific substrates and link them to LC3-containing membranes [33]. Several core ferroptosis regulators have been identified as substrates of selective autophagy. For example, p62-mediated autophagic degradation of the iron exporter ferroportin has been reported to increase intracellular Fe^2+^ levels and promote ferroptosis [34]. Other types of selective autophagy can modulate ferroptosis by altering lipid availability and cellular metabolism [35]. In this context, our data reveal that dronedarone sensitizes cells to ferroptosis by promoting p62-mediated autophagic degradation of GPX4. Dronedarone treatment enhanced the formation of GPX4-p62 complexes as evidenced by co-IP, and increased their colocalization by confocal microscopy, which is consistent with p62-dependent targeting of GPX4 for autophagic degradation. In line with these protein-level observations, scRNA-seq correlation analysis revealed a positive association between p62 and GPX4 in malignant cell clusters, further supporting a functional link between p62 and GPX4 regulation in pancreatic cancer. To further substantiate this, genetic evidence from p62 depletion experiments showed that knockdown of p62 substantially attenuated dronedarone-induced GPX4 degradation, supporting a p62-dependent mechanism. Under basal conditions, autophagic flux is maintained at homeostatic levels and GPX4-p62 colocalization is limited, allowing GPX4 to preserve cellular redox balance. Upon dronedarone exposure, autophagy is activated and GPX4-p62 interaction is enhanced, shifting this balance toward increased GPX4 degradation and enhanced ferroptosis.

Our mechanistic experiments further indicate that dronedarone exerts a dual impact on GPX family enzymes and cellular redox balance. A previous screening study focusing on GPX1 reported that dronedarone has relatively high binding affinity and inhibitory potential toward GPX1 compared with control compounds and their original targets [36]. Based on this observation, we used molecular docking and SPR to demonstrate a direct interaction between dronedarone and GPX4, accompanied by inhibition of GPX4 enzymatic activity. Molecular docking identified K162 and R179 as key residues in the interaction between dronedarone and GPX4. Site-directed mutagenesis and functional assays provide evidence that K162 likely serves as a critical site for dronedarone-induced ferroptosis, while R179 regulates basal enzymatic activity, potentially contributing to the overall cellular response. Thus, dronedarone promotes autophagy-dependent degradation of GPX4 and directly impairs its peroxidase activity, ultimately leading to cell death in pancreatic cancer cells.

Some limitations of this study should be noted. Given dronedarone’s multiple biological activities, our data support ferroptosis as a major component in our experimental setting, but contributions from other cell-death pathways cannot be excluded. Future studies are warranted to define the relative contributions and interactions among these cell-death pathways in different experimental and disease contexts. Regarding translational relevance, the concentrations of dronedarone used in this study are higher than those typically achievable in clinical settings. However, effective drug concentrations in vitro often exceed those achievable in the plasma due to differences in drug delivery, tissue distribution, and local concentrations in tumor microenvironments [37]. Future pharmacokinetic studies will be necessary to address this gap. Given dronedarone’s potential in targeting ferroptosis and its broad biological effects, enhancing its tumor selectivity will be crucial for maximizing therapeutic outcomes. Potential optimization strategies include a prodrug approach or the development of tumor-targeted delivery systems, such as nanoparticles or antibody-drug conjugates. Recent studies have highlighted the development of prodrugs that release the active compound selectively within the tumor microenvironment, improving both efficacy and reducing systemic toxicity [38]. Further investigation is needed to fully evaluate the translational potential of dronedarone-based ferroptosis therapy.

## Conclusion

In summary, our work identifies dronedarone as a previously unrecognized ferroptosis inducer in pancreatic cancer. Dronedarone directly binds to GPX4, inhibits its enzymatic activity and promotes its autophagy-dependent degradation through a p62-mediated pathway, thereby increasing the ferroptosis sensitivity of pancreatic cancer. Moreover, dronedarone exhibited potent antitumor effects in mouse pancreatic cancer models without obvious cardiotoxicity or major organ damage at effective doses. Our research provides a mechanistic foundation and translational rationale for repurposing dronedarone as a potential strategy for ferroptosis-based therapy in pancreatic cancer.

## Supplementary Information

Supplementary Material 1.

Supplementary Material 2.

Supplementary Material 3.

Supplementary Material 4.

Supplementary Material 5.

Supplementary Material 6.

Supplementary Material 7.

Supplementary Material 8.

Supplementary Material 9.

## Data Availability

Single-cell data after integration are available upon request to corresponding authors.

## Abbreviations

FDA: Food and Drug Administration
ROS: Reactive oxygen species
GPX4: Glutathione peroxidase 4
GSH: Glutathione
CCK-8: Cell Counting Kit-8
Nec-1: Necrostain-1
CQ: Chloroquine
Rapa: Rapamycin
Fer-1: Ferrostain-1
NSA: Necrosulfonamide
DSF: Disulfiram
MRI: Magnetic resonance imaging
KPC: LSL-G^12D/+ 1^, LSL-Trp53^R172H/+^, and Pdx1-Cre
scRNA-seq: single-cell RNA sequencing
IHC: Immunohistochemistry
DAB: 3,3'-diaminobenzidine
DEGs: Differentially expressed genes
FDR: Adjusted *P* value
ECL: Enhanced chemiluminescence
TEM: Transmission electron microscopy
MMP: Mitochondrial membrane potential
UMAP: Uniform manifold approximation and projection
shRNA: Short hairpin RNA
siRNA: Small interfering RNA
WT: Wild-type
SDF: Structure data file
PDB: Protein Data Bank
SPR: Surface plasmon resonance
IP: Immunoprecipitation
IF: Immunofluorescence
HTS: High-throughput screening
PCA: principal component analysis
KEGG: Kyoto Encyclopedia of Genes and Genomes
GO: Gene Ontology
