# Supplementary figures and images for: Repurposing dronedarone induces ferroptosis through GPX4 inactivation and degradation in pancreatic cancer
Source: J Exp Clin Cancer Res. 2026 Mar 14;45:102. doi: 10.1186/s13046-026-03687-6 (PMC13101247; doi:10.1186/s13046-026-03687-6)

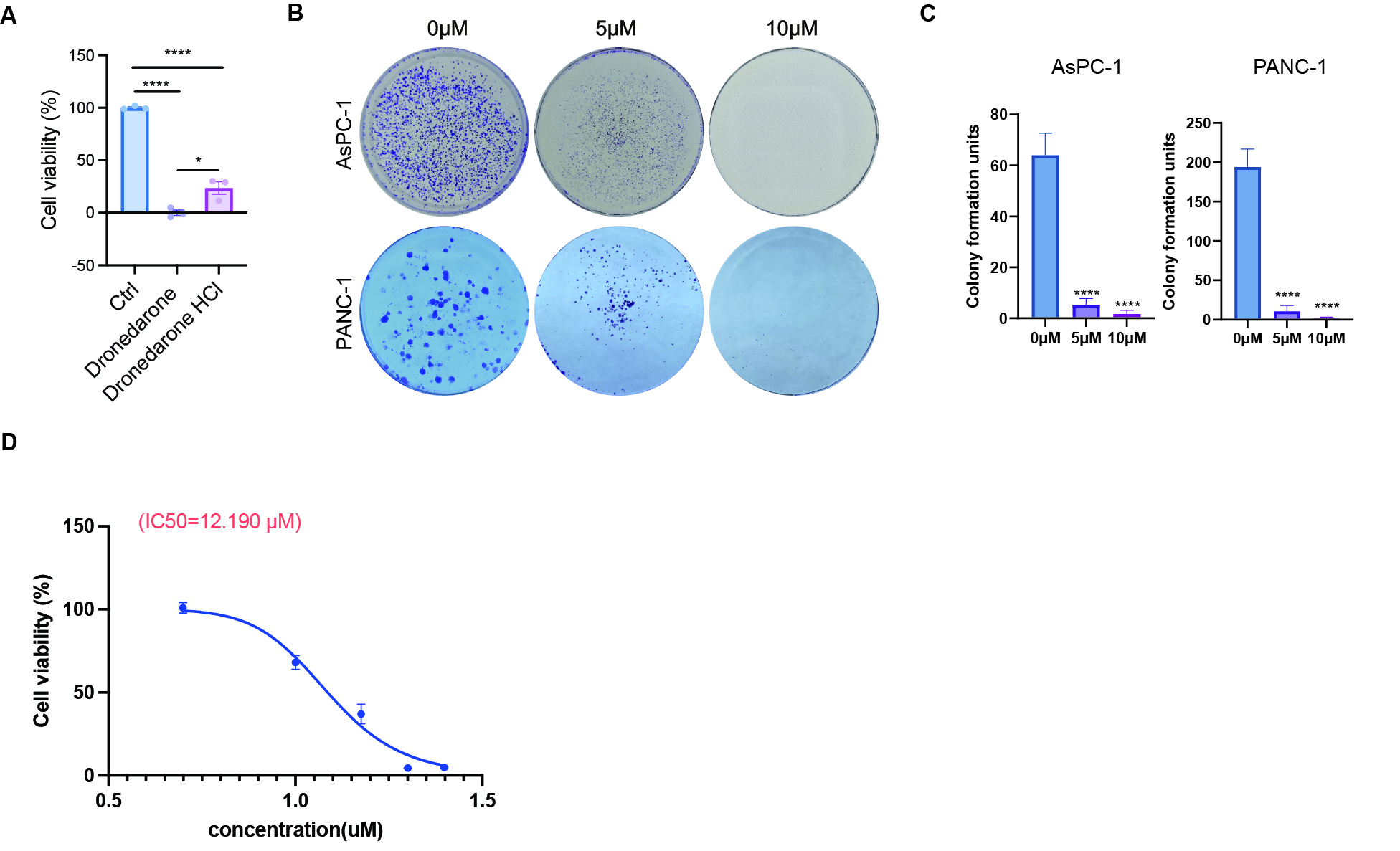

Supplement: Supplementary file 3 — Supplementary Material 3. [file 13046_2026_3687_MOESM3_ESM.jpg]

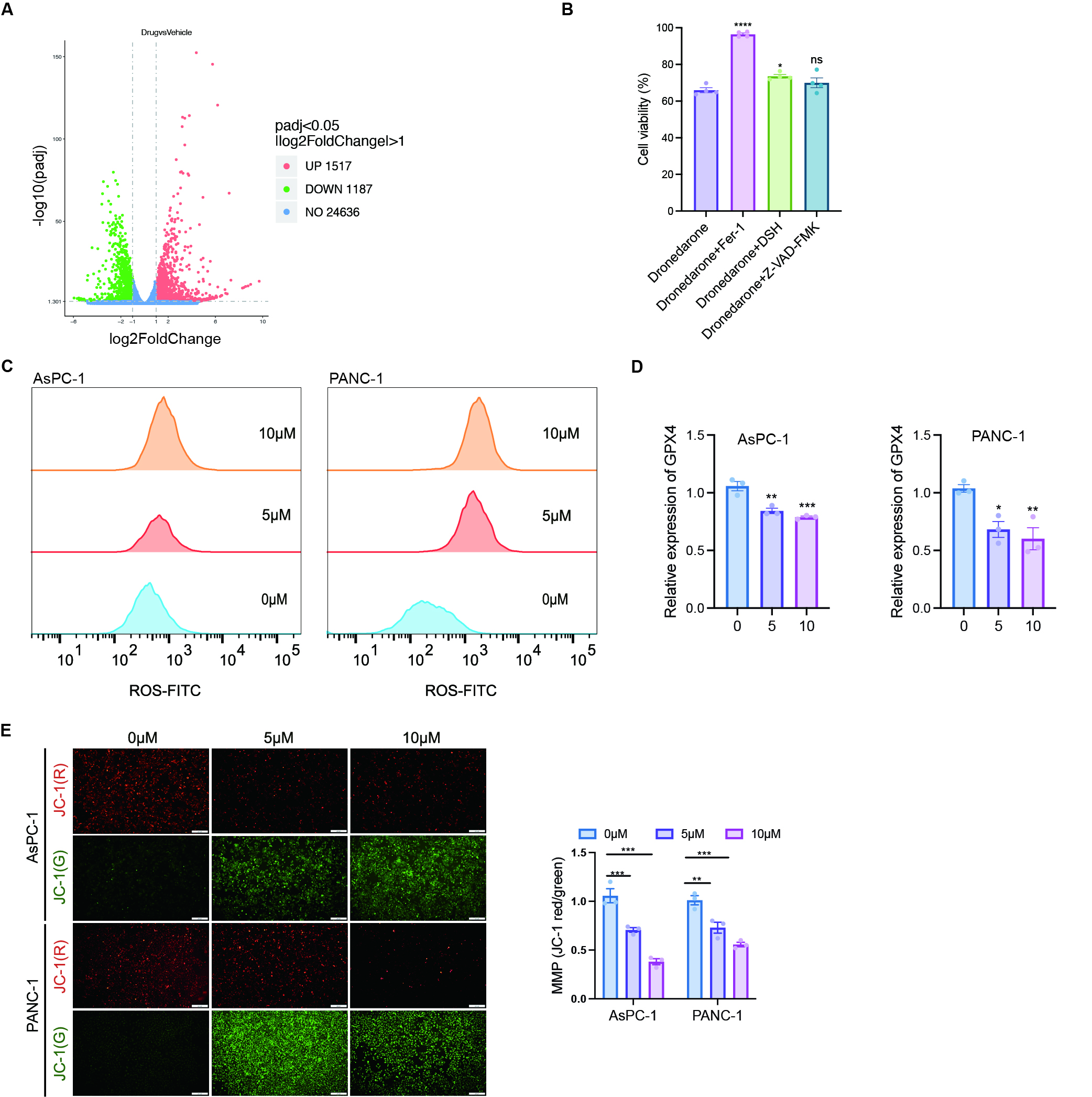

Supplement: Supplementary file 4 — Supplementary Material 4. [file 13046_2026_3687_MOESM4_ESM.jpg]

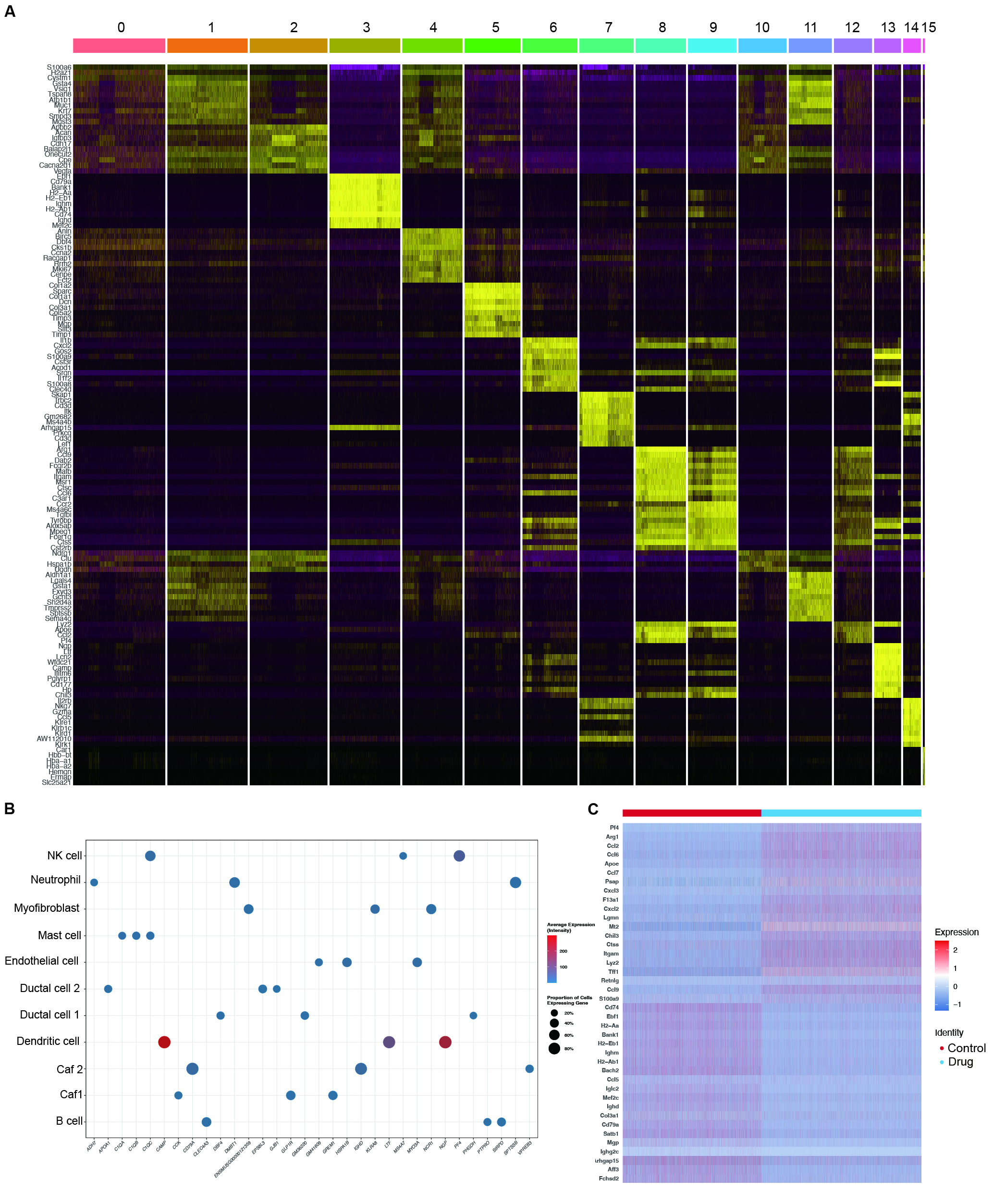

Supplement: Supplementary file 5 — Supplementary Material 5. [file 13046_2026_3687_MOESM5_ESM.jpg]

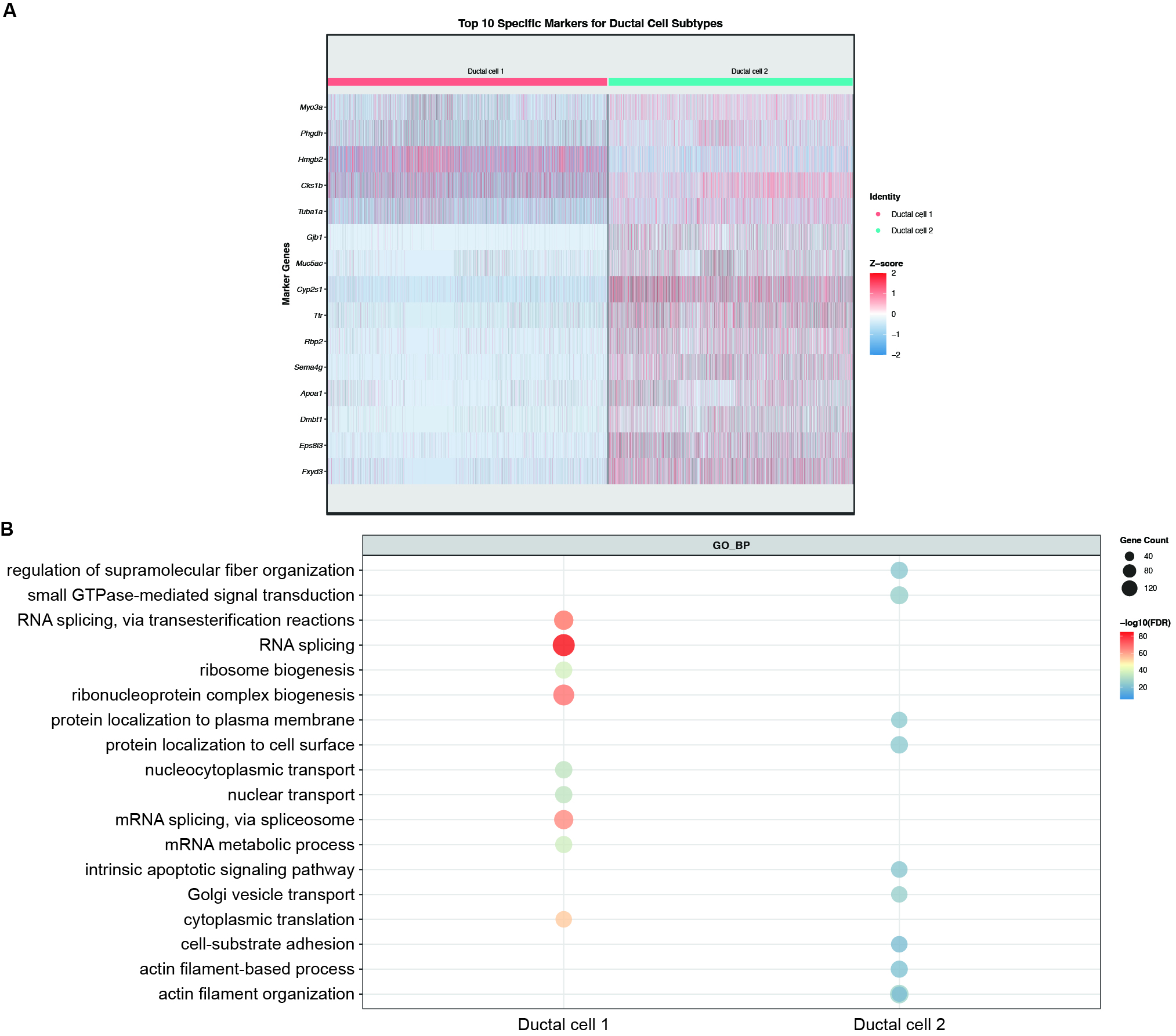

Supplement: Supplementary file 6 — Supplementary Material 6. [file 13046_2026_3687_MOESM6_ESM.jpg]

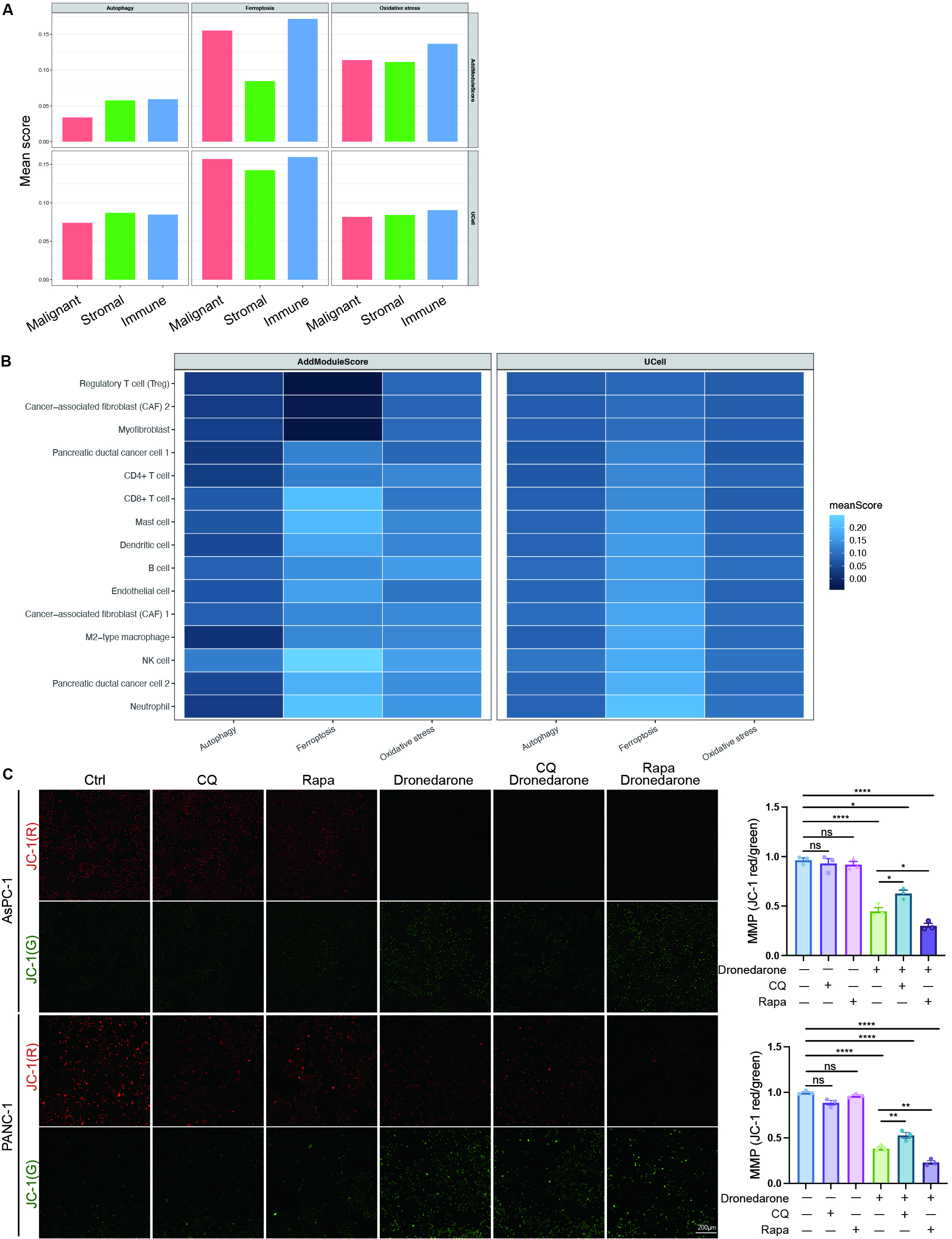

Supplement: Supplementary file 7 — Supplementary Material 7. [file 13046_2026_3687_MOESM7_ESM.jpg]

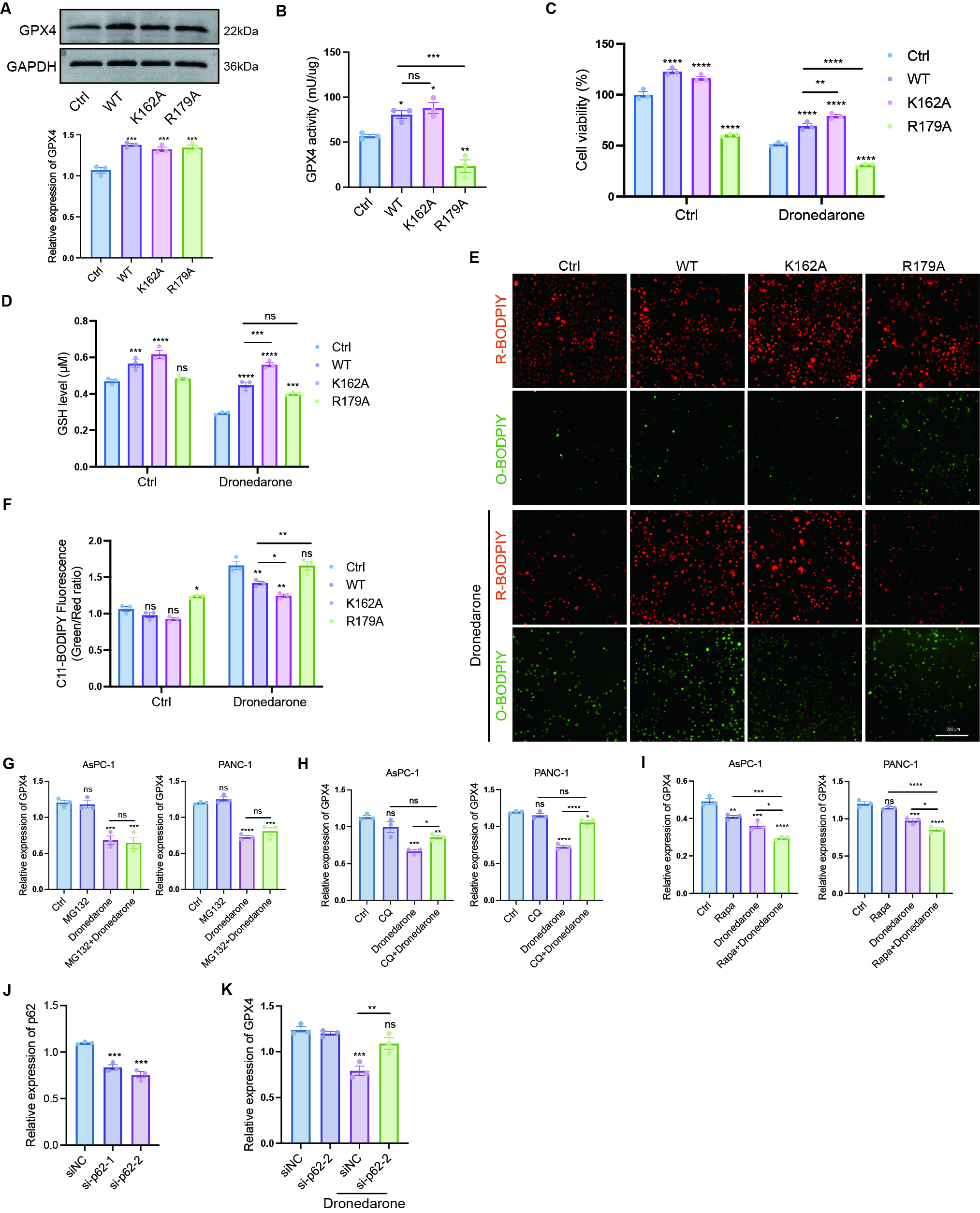

Supplement: Supplementary file 8 — Supplementary Material 8. [file 13046_2026_3687_MOESM8_ESM.jpg]

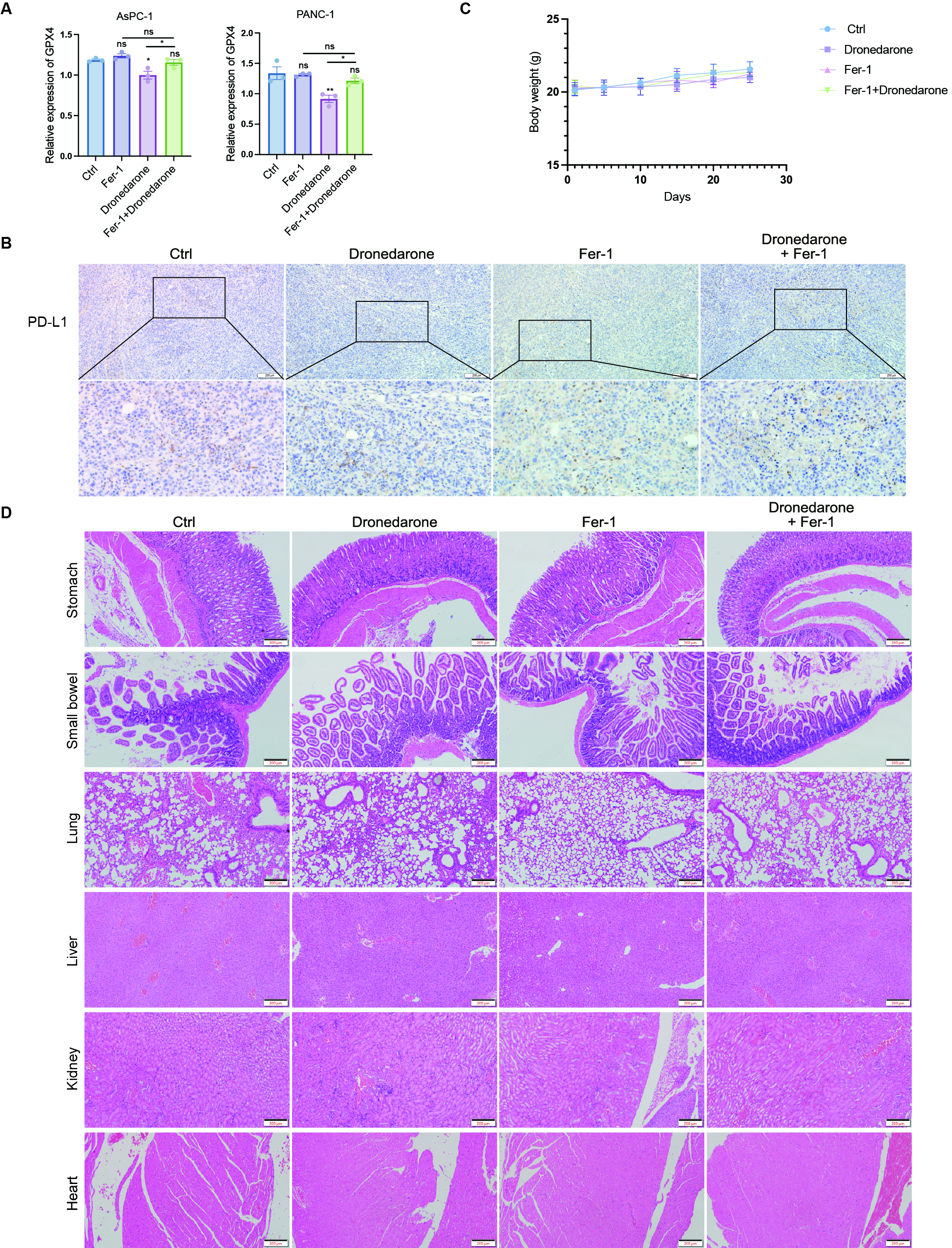

Supplement: Supplementary file 9 — Supplementary Material 9. [file 13046_2026_3687_MOESM9_ESM.jpg]
